# Hybrid extracellular vesicles drive irreversible mitochondria damage and TCA metabolite deficiency-related chondrocyte senescence

**DOI:** 10.1016/j.xinn.2025.101247

**Published:** 2026-01-05

**Authors:** Ting Xiang, Rong Zhang, Xuanyi Li, Xin Li, Jinyang Wang, Jiaqi Li, Yongxi Lu, Chi Zhang, Shangbin Zhang, Lili Chen, Qingbin Zhang, Xiaoxing Kou

**Affiliations:** 1 Hospital of Stomatology, Guanghua School of Stomatology, Sun Yat-sen University, Guangzhou Provincial Key Laboratory of Stomatology, Guangzhou 510055, China; 2 South China Center of Craniofacial Stem Cell Research, Hospital of Stomatology, Sun Yat-Sen University, Guangzhou 510055, China; 3 Department of Temporomandibular Joint, School and Hospital of Stomatology, Guangzhou Medical University, Guangzhou 510180, China; 4 Guangdong Engineering Research Center of Oral Restoration and Reconstruction, Guangzhou Medical University, Guangzhou 510180, China; 5 Department of Joint Surgery, The Third Affiliated Hospital, Guangzhou Medical University, Guangzhou 510150, China

**Keywords:** osteoarthritis, hybrid extracellular vesicles, metabolism, cartilage regeneration

## Abstract

Metabolic and inflammatory stresses play crucial roles in osteoarthritis (OA). However, the reasons behind the difficulty in correcting impaired metabolism and cellular dysfunction in OA chondrocytes remain unclear. Given the metabolic modulation effect of extracellular vesicles (EVs), we asked whether endogenous EVs play a critical role in OA. Here, we identified a subtype of hybrid extracellular vesicles (hEVs) enriched in the joint fluid from OA patients, correlating with OA severity. These hEVs exhibited dual markers from proinflammatory macrophages and chondrocytes, promoting chondrocyte uptake and enhancing metabolic regulatory capability. hEV administration increased chondrocyte damage, characterized by enhanced mitochondrial defects and cellular aging in OA models. Unlike the reversible metabolic damage induced by inflammation in cartilage stem/progenitor cells (CSPCs), hEVs induced irreversible mitochondrial fragmentation and sustained cellular aging, even after stimulus removal. Notably, while hEVs promoted a metabolic shift toward glycolysis in OA CSPCs, blocking glycolysis alone failed to restore CSPC dysfunction. Mechanistically, hEVs decreased the mitochondrial membrane potential (ΔΨm) in an ADP/ATP translocase 1 (ADT1)-dependent manner, contributing to irreversible mitochondria fragmentation. As a result, hEVs depleted tricarboxylic acid metabolites, particularly acetyl-CoA and α-ketoglutarate (α-KG), associated with altered histone acetylation and methylation in OA CSPCs. Thus, combination therapy with an ADT1 inhibitor, supplemented with acetyl-CoA and α-KG, corrected hEV-induced metabolic reprogramming and cellular fate changes, restoring impaired chondrogenesis and aging in OA CSPCs and OA models. This study reveals hEVs as unrecognized OA pathogenic drivers, linking EV-mediated irreversible mitochondria damage to chondrocyte aging, and opens a new avenue for OA treatment.

## Public summary

• Identifying hybrid extracellular vesicles in the joint fluid from osteoarthritis patients.
• Hybrid extracellular vesicles aggravates chondrocyte damage in osteoarthritis models.
• Hybrid extracellular vesicles drive irreversible mitochondria fragmentation and sustained cellular aging.
• Developing metabolically targeted combination strategies to treat osteoarthritis.

## Graphical abstract

**Figure:**
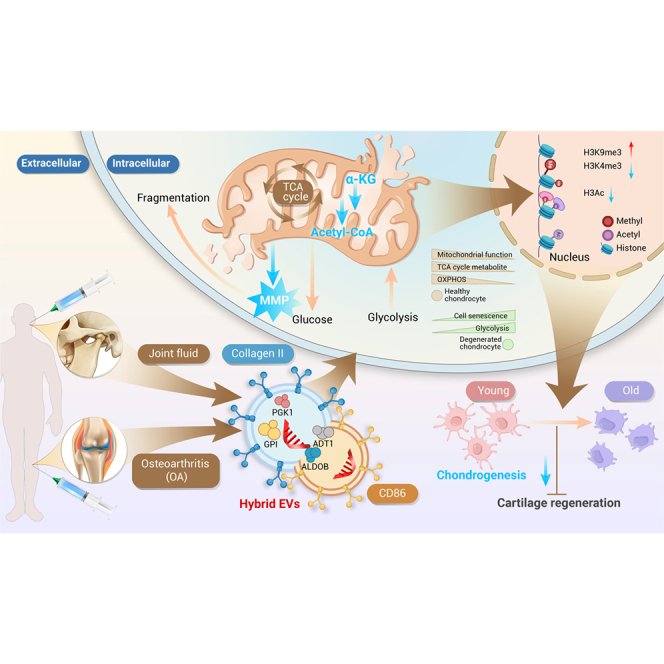

## Introduction

Disrupted metabolic homeostasis and inflammatory stimuli are key factors in the progression of various degenerative diseases, including osteoarthritis (OA).^1^ OA is one of the predominant disabling conditions, characterized by cartilage damage that fails to regenerate, affecting billions of people worldwide.^2^ Therefore, enzymes involved in metabolic pathways, metabolites, and inflammatory pathways may serve as potential therapeutic targets to treat OA.^3^^,^^4^^,^^5^^,^^6^ However, current treatments to effectively restore the metabolic imbalance and promote the regeneration of damaged chondrocytes in OA have not been validated.^7^^,^^8^ Therefore, understanding the underlying causes of irreversible metabolic changes is essential for uncovering the specific mechanisms contributing to limited regenerative capacity and developing effective strategies to treat OA.

Mitochondria and their tricarboxylic acid (TCA) cycle play crucial roles in regulating various cellular functions and determining cell fate.^9^ Intracellular metabolism is mediated by surrounding extracellular signals, such as inflammatory stress.^10^^,^^11^ However, despite inflammation, the endogenous factors affecting TCA-cycle-related metabolic networks remain largely unknown. Extracellular vesicles (EVs) are membrane-derived vesicles that play pivotal roles in intercellular communication and function regulation.^12^ Endogenous EVs can be detected in various body fluids,^13^ but the detailed functions of these humoral EVs in metabolic regulation and degenerative diseases are largely unknown. Specifically, the properties of joint-fluid-derived EVs and their function in OA progression remain elusive. Previous studies focused on the EVs originating from a single population of resident cells or immune cells infiltrating arthritic regions.^14^ Based on the evidence, EV membranes can fuse with target cell membranes, and EVs can be modified artificially by fusion with liposomes *in vitro*.^12^^,^^15^ We proposed that a subpopulation of hybrid EVs (hEVs) from different cell types may be enriched in the joint fluid of OA patients. As infiltrated proinflammatory immune cells and resident chondrocytes adapt to distinct metabolic pathways for their respective function, host cells are more liable to take up EVs that incorporate their own cell membranes.^16^^,^^17^ Therefore, if such hEVs exist in joint fluid, likely originating from both chondrocytes and immune cells, they may contribute to the dramatic metabolic changes and the failure of cartilage regeneration in OA.

The superficial layer of the cartilage contains a group of cartilage stem/progenitor cells (CSPCs) expressing stem cell markers and possessing high proliferation capacity and chondrogenic potential.^18^ These cells are present in healthy and OA cartilage and are recognized as key regulators in maintaining joint homeostasis and facilitating the repair process of cartilage.^19^^,^^20^ Unfortunately, these progenitor-like cells fail to repair OA cartilage defects efficiently.^7^ Therefore, it is strongly suggested that some as yet uncovered endogenous reasons halt the chondrogenic capacity of CSPCs in OA. Mitochondria are signaling organelles that not only play a central role in regulating cellular function through energy metabolism but also direct cell fate and affect aging through TCA-cycle metabolite-mediated mitochondrial-nuclear communication.^21^ Thus, we hypothesized that hEVs may affect the cell fate of CSPCs in OA through modulation of mitochondrial and TCA metabolism.

In this study, we identified that patients with accumulated joint fluid hEVs displayed severe OA features. hEVs induced irreversible mitochondrial damage in OA CSPCs in an ADP/ATP translocase 1 (ADT1)-dependent manner. The consequent reduction in TCA-cycle metabolites was responsible for the altered epigenetic modifications, linking to deficient chondrogenesis and senescence of CSPCs. Therefore, a combination therapy based on these metabolic targets, along with an ADT1 inhibitor, restored the mitochondrial damage and epigenetic changes, thus rescuing impaired chondrogenesis and aging of OA CSPCs and improving outcomes in OA models. Our findings revealed that these joint fluid EVs contribute to the irreversible metabolic changes and limited regenerative capacity of cartilage and paved the way for developing metabolically targeted therapies for OA.

## Materials and methods

The detailed methods for OA model, biodistribution of EVs, cell cultures, multilineage differentiation analysis, characterization of EVs (including nanoparticle tracking analysis [NTA], transmission electron microscopy [TEM], and nano-flow cytometry analysis), western blotting, immunofluorescence staining, flow cytometry analysis, proteomic/metabolomics/RNA-sequencing (RNA-seq) analysis, and RT-qPCR are provided in the supplemental information.

### Animals

With the approval of the ethics committees of Sun Yat-Sen University (SYXK-2024-0081), a total of 94 female Sprague-Dawley rats (180–200 g) were randomly assigned to either the experimental (*n* = 54) or control (*n* = 40) groups. All rats were housed on a 12-h light/12-h dark cycle under controlled temperature (22°C ± 1°C) and had free access to food and water.

### Patient samples

**Figure 1:**
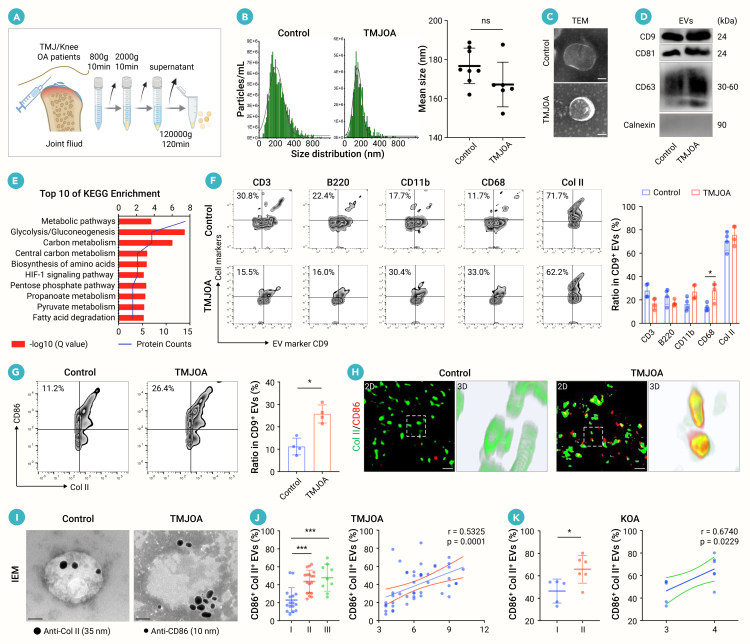
Identification of hybrid EVs in the joint fluid of OA patients and their correlation with disease progression (A) Schematic diagram illustrating the isolation procedure for joint fluid EVs. (B) Size distribution of joint fluid EVs analyzed by nanoparticle tracking analysis (NTA; *n* = 6–8). (C) Representative transmission electron microscopy (TEM) images showing EV morphology. Scale bar, 100 nm. (D) Western blotting analysis of EV-positive markers (CD9, CD81, and CD63) and negative marker calnexin in EVs isolated from control and TMJOA patients. (E) Top ten enriched KEGG pathways for differentially expressed genes (DEGs) in joint fluid EVs from TMJOA patients versus controls. (F and G) Nano-flow cytometry analysis showing the hybrid cellular orientation of the EVs from joint fluid. Quantifications showing the proportions of CD9^+^CD68^+^, CD9^+^collagen II^+^, and CD86^+^collagen II^+^ double-positive EVs in control and TMJOA patient joint fluid (*n* = 4). (H) Representative confocal microscopy images demonstrating that an increased number of hybrid EVs (hEVs) expresses both M1 macrophage marker CD86 (red) and chondrocyte marker collagen II (green). Magnified 3D images of the boxed regions are shown. Scale bar, 5 μm. (I) Immunoelectron microscopy (IEM) of hEVs dual labeled with anti-collagen II (35-nm gold particles) and anti-CD86 (10-nm gold particles). Scale bar, 100 nm. (J and K) Percentage of hEVs in TMJOA (J) or KOA (K) patients stratified by disease stage, and correlation between hEV levels and disease severity. TMJOA, *n* = 43 patients; KOA, *n* = 11 patients. Data represent mean ± SD. ∗*p* < 0.05 by *t* test (B, G, and K). ∗*p* < 0.05 and ∗∗∗*p* < 0.001 by one-way ANOVA with Tukey’s test (F and J).

Samples of joint fluid from 41 patients undergoing joint fluid irrigation therapy (females and males aged 14–55 years) were collected, including 29 patients diagnosed with temporomandibular joint OA (TMJOA) and 12 control patients with disk displacement and no pain symptoms.^22^ The severity of TMJOA changes was evaluated with a clinical dysfunction index (Di).^23^ Specifically, the scores of the four items in “range of mandibular motion” are defined as standardized scores of 0 (0 points), 1 (1–4 points), and 5 (5–10 points), respectively. These standardized scores are then added to the scores of the other four clinical symptoms (TMJ function impairment, muscle tenderness during palpation, TMJ pain during palpation, and pain during mandibular movement) to obtain the Di score. The obtained Di scores were respectively standardized as I (1–4 points), II (5–9 points), and III (10–25 points). Meanwhile, joint fluid was collected from 17 knee joint OA (KOA) patients (females and males aged 45–75 years) who underwent drug injection therapy or total knee replacement surgery and had no systemic diseases, infections, or rheumatoid arthritis. The severity of KOA was assessed based on the Kellgren-Lawrence score of X-ray films.^24^ The study was approved by the Institutional Review Board of The School of Stomatology Affiliated to Guangzhou Medical University (JCYJ2024027) and the Institutional Review Board of The Third Affiliated Hospital of Guangzhou Medical University (JCYJ2024027 and YLHS2025029). All joint fluid samples were collected during clinical visits, after which they were immediately taken to a laboratory to isolate the EVs (Figure 1A).

### Preparation and characterization of joint fluid EVs

As described in the literature,^25^ joint fluid EVs were isolated by differential centrifugation. For knee joint fluid, we used hyaluronidase (Sigma, H3506) to reduce viscosity and obtain homogenized joint fluid before EV separation and characterization as suggested.^13^ In brief, samples of joint fluid were subjected to centrifugation at 800 × *g* and 2,000 × *g* (4°C for 10 min), and the pellet was removed. Subsequently, supernatants were spun for 120 min at 120,000 × *g* and 4°C in an SW41Ti rotor (Beckman Coulter, Brea, CA) to pellet the EVs.

### Measurement of acetyl-CoA and α-KG

We measured acetyl-coenzyme A (CoA) activity in CSPCs using an Acetyl-CoA Content Assay Kit (Solarbio) following the manufacturer’s instructions. α-Ketoglutarate was measured in CSPCs using the α-KG Content Assay Kit (Solarbio) following the manufacturer’s instructions.

### Combination therapy

For the combination therapy, the rats received an injection of combined drugs (bongkrekic acid [BKAl 120 μg/kg] + acetate [0.5 g/kg] + α-KG [10 mg/kg]) 1 week after the establishment of the OA model. At the endpoint, rats were sacrificed to collect TMJ and knee joint samples for further analysis, as shown in Figure 7A. As for CSPCs, 24 h after stimulation with interleukin-1β (IL-1β), either alone or in combination with *in vitro* hEVs (*iv*-hEVs), the culture medium was replaced with complete medium supplemented with BKA (0.1 μM/mL), acetate (0.2  mM/mL), and α-KG (1.41 mg/mL), followed by an additional 24-h incubation.

### Statistics

All data are presented as mean ± SD. Statistical and figure analyses were performed using GraphPad Prism 9 software. Comparisons between two groups were analyzed using independent two-tailed Student's *t* tests, while comparisons involving more than two groups were analyzed using one-way ANOVA. *p* < 0.05 was considered significant.

## Results

### Characterization of hEVs accumulated in the joint fluid of OA patients

To explore the impact of joint fluid EVs on disease progression in OA patients, we isolated EVs from the joint fluid of patients with TMJOA and KOA (Figure 1A), which involve the major joints affected by OA. NTA revealed that EVs from TMJOA and control individuals displayed an average size of 167.2 and 176.8 nm with similar membrane potential (Figures 1B and S1A). TEM and western blotting analysis showed that these joint fluid EVs had a spherical structure and expressed EV markers CD9, CD81, and CD63 while being negative for the large EV marker calnexin (Figures 1C and 1D). Liquid chromatography-tandem mass spectrometry analysis showed the enrichment of three categories of proteins in joint fluid EVs (Figure S1B), consistent with the guidelines outlined in MISEV2023.^13^ Further analysis of the enriched proteins in EVs from TMJOA patients using Kyoto Encyclopedia of Genes and Genomes (KEGG) pathways analysis revealed that these proteins are primarily involved in metabolic regulation (Figure 1E).

Joint fluid EVs from OA patients may originate from the resident joint cells and the infiltrating immune cells.^26^ Chondrocytes adapt to oxidative phosphorylation (OXPHOS), while proinflammatory immune cells primarily utilize glycolysis for differentiation. Thus, the origin of OA joint fluid-derived EVs from these parent cells may influence their unique metabolic regulating abilities. We used nano-flow cytometry to test our hypothesis and showed that over 70% of the CD9^+^ EVs from both control individuals and TMJOA patients were positive for the chondrocyte marker collagen II (Figures 1F and S1C–S1F). When we checked the surface markers of major immune cells associated with OA,^27^ we found that these joint fluid EVs were positive for markers from T cells, B cells, monocytes, and macrophages. Notably, only CD68^+^CD9^+^ EVs derived from macrophages were increased in joint fluid from OA patients (Figures 1F and S1F). Another intriguing observation was that the total positivity ratios of EVs originating from various cell types exceeded 100%, suggesting the expression of multiple cell markers by a single EV.

Given that most EVs in joint fluid express chondrocyte markers and that there is an increased presence of macrophage markers in EVs from OA patients, we further investigated the dual expressions of chondrocyte and immune cell markers in these EVs. As expected, we observed an increased subset of EVs co-expressing collagen II and the proinflammatory macrophage marker CD86 in the joint fluid of OA patients. In contrast, no significant changes were detected in the EVs co-expressing collagen II with other immune cell markers (Figures 1G and S1G). Consequently, we identified a specific subset of collagen II^+^CD86^+^ EVs, termed hEVs, enriched in the joint fluid of OA patients. To further verify the presence of hEVs, we used immunofluorescence staining, analyzed by super-resolution structured illumination microscopy (SIM) and 3D reconstruction, to demonstrate the increase in dual expression of collagen II^+^CD86^+^ in the EVs from OA patients (Figure 1H). Additionally, immunoelectron microscopy validated the dual surface localization of collagen II (35-nm gold particles) and CD86 (10-nm gold particles) on these hEVs (Figure 1I). Thus, our results support the notion that hEVs exist in joint fluid, which may play a role in the pathology of OA.

EVs isolated from KOA patients showed characteristics similar to those from TMJOA, which displayed an average size of around 170 nm and expressed EV markers CD9, CD81, and CD63 while being negative for calnexin (Figures S1H and S1I). Critically, the collagen II^+^CD86^+^ hEVs showed a gradual accumulation in the joint fluid of patients with severe OA in both the TMJOA and KOA cohorts (Figures 1J, 1K, S1J, and S1K; Tables S1 and S2). Correlation analysis confirmed a positive association between the levels of hEVs and the severity of OA in both the TMJOA (*n* = 43, *r* = 0.5325, *p* = 0.0001) and KOA (*n* = 11, *r* = 0.6740, *p* = 0.0229) cohorts.

### hEVs exacerbate OA pathogenesis and aggravate cartilage degradation with damaged mitochondria of chondrocytes in rat models

**Figure 2:**
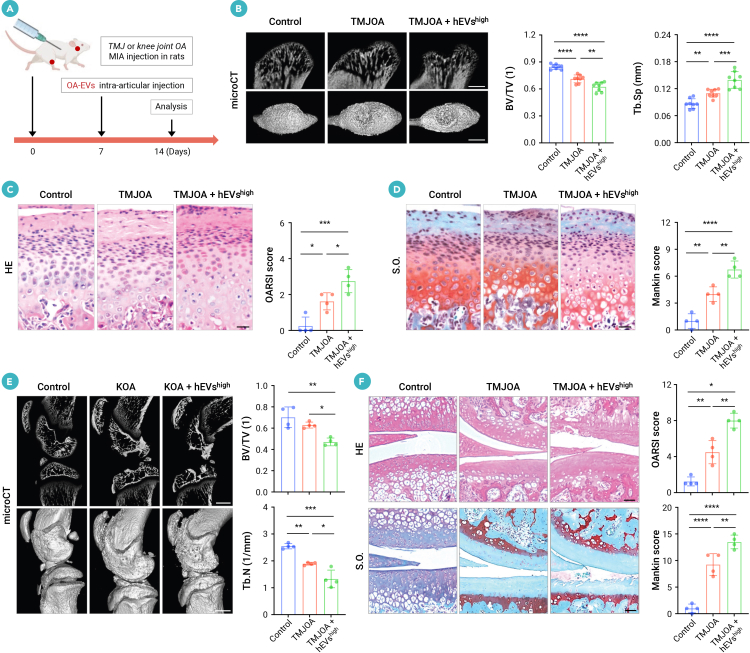
Intra-articular injection of hEVs exacerbates OA progression and impairs chondrogenesis in rat OA models (A) Scheme illustrating hEV intra-articular injection procedure of TMJOA/KOA rat models. (B) Micro-CT images and quantitative analysis of subchondral bone parameters in the condylar sagittal plane (*n* = 4). Scale bar, 1 mm. (C and D) Hematoxylin & eosin (H&E) (C) and safranin O (SO) (D) staining and histopathological scores (Osteoarthritis Research Society International [OARSI] and Mankin) for condylar cartilage (*n* = 4). Scale bar, 200 μm. (E) Micro-CT images and subchondral bone quantification of knee joints (*n* = 4). Scale bar, 1 mm. (F) H&E and SO staining and histopathological scores (OARSI and Mankin) for knee cartilage (*n* = 4). Scale bar, 200 μm. Data represent mean ± SD. ∗*p* < 0.05, ∗∗*p* < 0.01, ∗∗∗*p* < 0.005, and ∗∗∗∗*p* < 0.0001 by one-way ANOVA with Tukey’s test (B–F). hEVs^high^, EVs derived from OA joints; hEVs^low^, EVs derived from healthy joints.

Next, we evaluated the pathogenicity of hEVs in sodium iodoacetate-induced rat OA models by intra-articular injection of EVs isolated from the joints of OA and control rats, respectively (Figure 2A). First, we detected an increased level of collagen II^+^CD86^+^ hEVs in the joints of OA rats, rising from 10.0% ± 1.1% to 42.6% ± 4.3% compared to the control group (Figure S2A), which is consistent with the hEVs observed in OA patients (see Figure 1G). The biodistribution of EVs is crucial for their effects, and we found that a large number of PKH26-labeled hEVs were detected in the superficial and middle zones of cartilage 2 h after intra-articular injection and gradually accumulated into the deep zone at 6 h post injection (Figure S2B). Micro-computed tomography (micro-CT) analysis showed that hEV^high^ (hEVs from OA joints, which contain a higher proportion of hEVs) injection led to pronounced condylar bone resorption compared to TMJOA rats (Figure 2B). We then examined the cartilage degradation in hEV^high^-injected rats using hematoxylin and eosin (H&E), safranin O-fast green (SO), and toluidine blue (TB) staining. We found that hEV^high^ injection further aggravated the progression of OA in TMJOA rats, as evidenced by increased fibrosis of the cartilage matrix and disrupted cellular distribution, decreased proteoglycan density, and diminished glycosaminoglycan content compared to TMJOA rats (Figures 2C, 2D, and S2C). Consistently, hEV^high^ injection also resulted in worsened progression of KOA in rats, as indicated by increased subchondral bone loss, aggravated cartilage degradation, and decreased chondrogenesis (Figures 2E, 2F, and S2D). Next, we evaluated the pathogenicity of hEVs in rat OA models by comparing intra-articular injections of hEV^high^ to those from healthy joints with a lower proportion of hEVs (hEV^low^) (Figure S3A). Compared to the aggravation effect of the hEV^high^ on OA progression, hEV^low^ did not exacerbate bone resorption and cartilage degradation in both TMJOA and KOA rats, as assessed by micro-CT, H&E, and SO analysis (Figures S3B–S3F). Together, these findings suggest that hEVs derived from OA joints contribute to the exacerbation of the OA pathogenesis in rats.

### hEVs display distinct functions on metabolic reprogramming compared to EVs from a single cellular source

**Figure 3:**
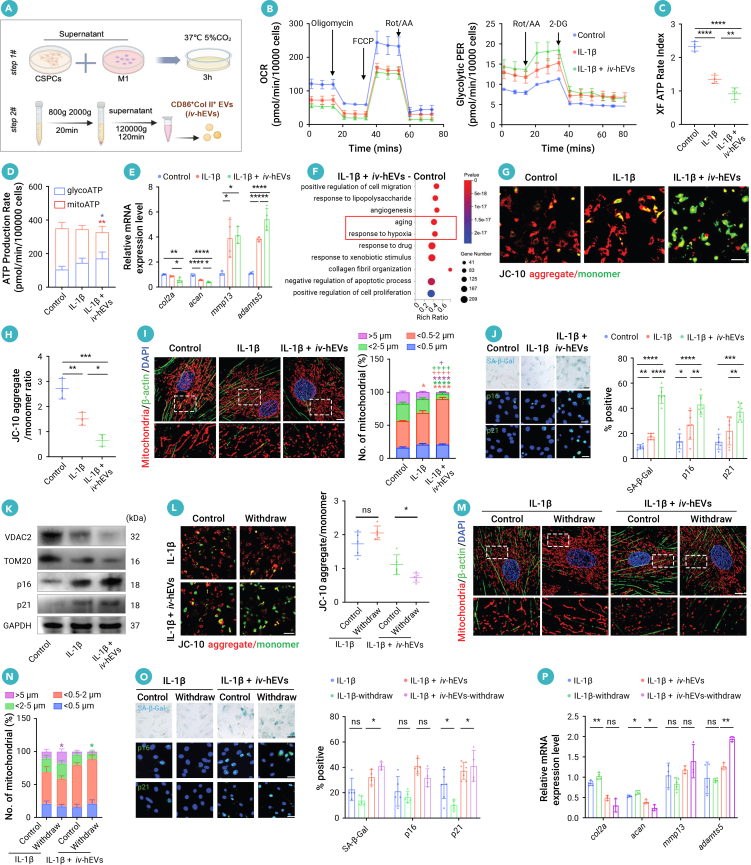
hEVs induce metabolic reprogramming and irreversible mitochondria fragmentation with cellular senescence in CSPCs (A) Schematic of *in vitro* hEV (*iv*-hEVs) generation. (B) Seahorse analysis of oxygen consumption rate (OCR) and glycolytic proton efflux rate (PER) in CSPCs (*n* = 4). (C and D) ATP production in CSPCs of indicated groups (*n* = 4). (E) RT-qPCR analysis of cartilage anabolic and catabolic genes (*n* = 3–4). (F) GO functional enrichment of DEGs in IL-1β + *iv*-hEVs-treated CSPCs versus controls. (G and H) JC-10 staining quantifying mitochondrial membrane potential (ΔΨm) of CSPCs (*n* = 3). Scale bar, 200 μm. (I) Representative confocal images and quantification of mitochondria fragmentation in CSPCs (*n* = 6). Magnified images of the boxed regions are shown in the lower panel. Scale bar, 5 μm. (J) SA-β-Gal, p16, and p21 staining and quantification of senescent CSPCs (*n* = 6–7). Scale bar, 100 μm. Control and CSPCs treated by IL-1β with or without *iv*-hEVs for 24 h were used in relative experiments (B–J). (K) Western blotting analysis of VDAC2, TOM20, p16, and p21. Protein expressions were detected in CSPCs 24 h post treatment with IL-1β or IL-1β + *iv*-hEVs. (L) JC-10 staining quantifying ΔΨm of CSPCs post treatment withdrawal (*n* = 5). Scale bar, 200 μm. (M and N) Representative confocal images and quantification of mitochondria fragmentation in CSPCs post treatment withdrawal (*n* = 6). Magnified images of the boxed regions are shown in the lower panel. Scale bar, 5 μm. (O) SA-β-Gal, p16, and p21 staining and quantification of senescent cells in CSPCs post treatment withdrawal (*n* = 4–7). Scale bar, 100 μm. (P) RT-qPCR analysis of cartilage anabolic and catabolic genes in CSPCs post treatment withdrawal (*n* = 3–5). CSPCs were treated by IL-1β with or without *iv*-hEVs for 24 h, followed by continuous or withdrawal of former treatment (L–P). Data represent means ± SD. ∗*p* < 0.05, ∗∗*p* < 0.01, ∗∗∗*p* < 0.001, and ∗∗∗∗*p* < 0.0001 versus control (C–E, H, J, L, O, and P); ∗*p* < 0.05, ∗∗∗∗*p* < 0.0001 versus control and ^+^*p* < 0.05, ^++++^*p* < 0.0001 versus IL-1β (I) by one-way ANOVA with Tukey’s test.

To mimic the hEVs from OA joint fluid and to explore the function of hEVs on chondrocytes, we incubated the cell-culture supernatant from rat M1 macrophages and IL-β-treated CSPCs, a widely used OA chondrocytes model *in vitro*,^28^ to generate hEVs from these two cellular sources (Figure 3A). First, we confirmed the characteristics of CSPCs by observing the positive expressions of MSC markers and negative expressions of hematological markers, as well as their significant osteogenic, adipogenic, and chondrogenic differentiation capacities (Figures S4A and S4B). Next, we evaluated the fusion rate of *iv*-hEVs using nano-flow cytometry. The results demonstrated that the proportion of *iv*-hEVs that were double positive for collagen II and CD86 gradually increased, peaking at 26% at 3 h post fusion (Figure S4C). Interestingly, SIM images and co-localization analysis revealed that CSPCs were more efficient than macrophage-derived EVs at taking up CSPC-derived EVs (Figure S4D). Conversely, a similar amount of macrophage and CSPC-derived signals carried by *iv*-hEVs were internalized by recipient CSPCs (Figure S4E), which indicates that *iv*-hEVs may facilitate the uptake of EV contents from macrophages.

We then compared the differences in CSPC metabolic regulation of single-cellular source-derived OA CSPC-EVs (C-EVs), M1 macrophage-EVs (M1-EVs), and *iv*-hEVs. Seahorse analysis revealed that *iv*-hEVs exhibited a more pronounced inhibitory effect on mitochondrial maximal respiration and significantly enhanced the glycolytic proton efflux rate (glycoPER) compared to untreated CSPCs (Figures S4F–S4H). Furthermore, ATP-rate assays showed decreased ATP production through mitochondrial OXPHOS in *iv*-hEV-treated CSPCs compared to other groups (Figure S4I). These findings suggest that hEVs possess a distinct ability for metabolic reprogramming of CSPCs.

### hEVs intensify inflammation-induced metabolic switch toward glycolysis and induce irreversible mitochondrial damage in OA CSPCs

We further examined the metabolic regulating effect of *iv*-hEVs on OA CSPCs treated by IL-1β. Analysis of mitochondrial respiration showed that mitochondrial ATP production decreased following IL-1β treatment, and *iv*-hEVs further inhibited mitochondrial ATP production in IL-1β-treated CSPCs. We also assessed the glycolysis function of CSPCs and found that compensatory glycolysis increased significantly after *iv*-hEV treatment (Figures 3B, S4J, and S4K). Additionally, *iv*-hEV-treated CSPCs exhibited a reduction in ATP production through mitochondrial OXPHOS, accompanied by an increase in ATP generation through glycolysis (Figures 3C and 3D). These findings indicate that *iv*-hEVs exacerbate the inflammation-induced metabolic switch toward glycolysis in CSPCs.

To investigate the effects of metabolic dysfunction in CSPCs induced by *iv*-hEVs, we performed RT-qPCR to evaluate the expression of cartilage anabolic and catabolic genes. Our findings showed that *iv*-hEVs further suppressed cartilage synthesis and enhanced cartilage degradation induced by IL-1β, as evidenced by further downregulation of type II collagen (*col2a1*) and aggrecan (*acan*), along with the upregulation of matrix metalloproteinase 13 (*mmp13*) and a disintegrin and metalloproteinase with thrombospondin motifs 5 (*adamts5*) (Figure 3E).

We then conducted RNA-seq with Gene Ontology (GO) analysis to show that CSPCs treated with a combination of IL-1β and *iv*-hEVs exhibited enrichment of gene sets related to “response to hypoxia” and “aging” (Figure 3F). Furthermore, the “response to hypoxia” gene set was also enriched when comparing CSPCs treated with IL-1β and *iv*-hEVs to those treated with IL-1β alone (Figure S4L). Based on these findings, along with the observation that *iv*-hEV treatment exacerbated IL-1β-induced repression of mitochondrial OXPHOS (Figures 3B and 3D), we proposed that *iv*-hEVs may intensify mitochondrial dysfunction in OA CSPCs.

Preserving normal ΔΨm is crucial for maintaining mitochondrial function.^29^^,^^30^ Thus, we used JC-10 staining to show that *iv*-hEV treatment reinforced the decrease of ΔΨm induced by IL-1β in CSPCs, as indicated by further increased monomers and decreased aggregates (Figures 3G and 3H). Consistently, SIM analysis of TOM20 staining showed that *iv*-hEV treatment exacerbated the mitochondrial fragmentation induced by IL-1β in CSPCs, indicated by further decreased percentage of long mitochondria and increased percentage of short mitochondria stimulated by IL-1β (Figure 3I). Since mitochondrial dysfunction is an important hallmark of aging^31^ and is linked to impaired chondrogenesis in OA,^32^ we examined several senescence markers and found that *iv*-hEVs notably enhanced the IL-1β-induced increase of SA-β-Gal-, p16-, and p21-positive cells (Figure 3J). These findings were consistent with the western blotting, and RNA-seq analyses also displayed an enriched “aging” profile in CSPCs treated with *iv*-hEVs (Figures 3F–3K and S4M). Additionally, *iv*-hEVs displayed a stronger capability to decrease ΔΨm, induce mitochondrial fragmentation, and elevate senescence markers when compared to EVs derived from single-cellular sources such as C-EVs and M1-EVs (Figures S5A–S5D). We next verified this effect *in vivo* and revealed that hEV^high^ injection further increased mitochondria fragmentation and decreased the number of mitochondria as well as elevating the levels of p16- and p21-positive senescent cells in chondrocytes within the proliferative layer of cartilage compared to rats with TMJOA and KOA (Figures S5E–S5G).

However, it remains unclear why OA CSPCs failed to repair. Inspired by the reinforced mitochondrial damage profile induced by *iv*-hEVs in IL-1β-treated CSPCs, we further investigated the repair capabilities of CSPCs after removing the stimuli. Interestingly, we found that the impaired CSPCs partially recovered from IL-1β-induced mitochondrial damage and the elevation of senescence markers following withdrawal of IL-1β stimulation (Figures 3L–3O). In contrast, the impaired CSPCs displayed even more severe mitochondrial damage and cellular senescence after withdrawal of combined stimulation from IL-1β and *iv*-hEVs, as indicated by the further reduced ΔΨm, decreased percentage of long mitochondria, and increased SA-β-Gal- and p21-positive cells (Figures 3L–3O). Consequently, the damaged CSPCs showed increased expression of *col2a* and *acan* after IL-1β withdrawal (Figure 3P). However, CSPCs failed to recover from the degenerative profile after the withdrawal of combined stimulation of IL-1β and *iv*-hEVs, as indicated by the downregulation of *acan* and upregulation of *adamts5* (Figure 3P). These findings suggest that *iv*-hEVs lead to irreversible mitochondrial damage and cellular senescence in OA CSPCs, which are linked to their limited chondrogenic capability.

### Solely glycolysis inhibition fails to rescue hEV-induced mitochondrial damage and CSPC dysfunction

**Figure 4:**
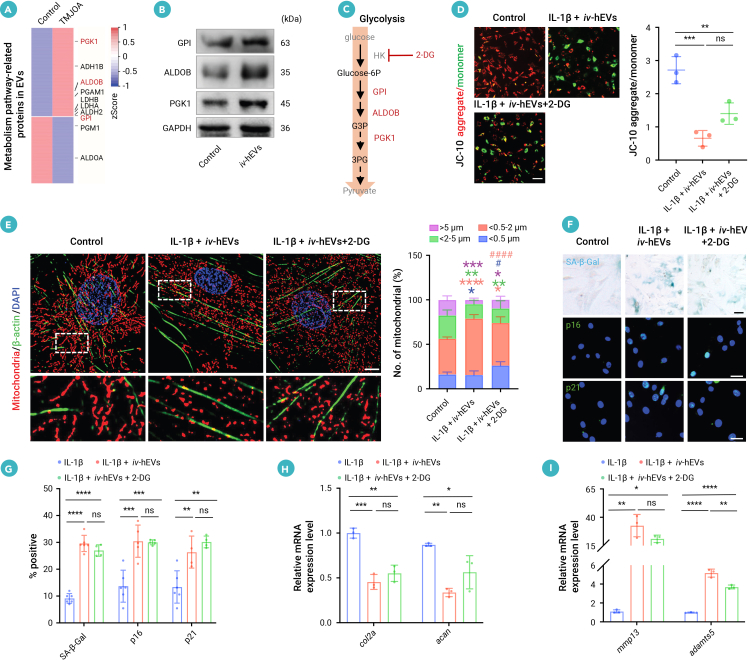
Glycolysis inhibition shows limited ability to restore hEV-induced CSPC damage (A) Heatmap of metabolism-related DEPs of joint fluid EVs from TMJOA patients versus controls. The upregulated proteins related to glycolysis, GPI, ALDOB, and PGK1 were labeled. (B) Western blotting analysis of glycolytic enzymes (GPI, ALDOB, and PGK1). Protein expressions were detected in CSPCs 24 h post treatment with *iv*-hEVs. (C) Schematic of *iv*-hEV-enriched glycolytic proteins and how glycolysis-related inhibitor 2-DG was used to treat CSPCs. (D) JC-10 staining quantifying ΔΨm in 2-DG-treated CSPCs (*n* = 3). Scale bar, 200 μm. (E) Representative confocal images and quantification of mitochondria fragmentation in CSPCs treated with IL-1β + *iv*-hEVs with or without 2-DG treatment (*n* = 5–6). Magnified images of the boxed regions are shown in the lower panel. Scale bar, 5 μm. (F and G) SA-β-Gal, p16, and p21 staining and quantification of senescent cells in 2-DG-treated CSPCs (*n* = 4–6). (H and I) RT-qPCR of cartilage anabolic and catabolic genes in 2-DG-treated CSPCs (*n* = 3). Data represent mean ± SD. ∗*p* < 0.05, ∗∗*p* < 0.01, ∗∗∗*p* < 0.005, ∗∗∗∗*p* < 0.0001 versus control and ^##^*p* < 0.01, ^####^*p* < 0.0001 versus IL-1β + *iv*-hEVs (E); ∗*p* < 0.05, ∗∗*p* < 0.01, ∗∗∗*p* < 0.005, and ∗∗∗∗*p* < 0.0001 (D, G–I) by one-way ANOVA with Tukey’s test.

Proteomic analysis of differentially expressed proteins (DEPs) in EVs from OA patients displayed an enriched profile of metabolic pathways (Figure 1E). We further analyzed the glycolysis-related proteins in OA EVs to identify potential candidates that might influence glycolysis in OA CSPCs. The result showed that EVs from OA patients highly expressed several glycolytic enzymes, including glucose-6-phosphate isomerase 1 (GPI), aldolase B (ALDOB), and phosphoglycerate kinase (PGK1) (Figure 4A). Next, we confirmed that *iv*-hEV treatment notably increased the levels of GPI, ALDOB, and PGK1 in CSPCs (Figure 4B), which coincided with the enhanced glycolysis in *iv*-hEV-treated CSPCs (Figures S4G and S4H). Thus, to investigate whether blocking glycolysis could reverse *iv*-hEV-induced mitochondrial damage in CSPCs, we used the glycolysis inhibitor 2-deoxyglucose (2-DG) to target the upstream rate-limiting enzyme hexokinase (HK) (Figure 4C). Unexpectedly, while 2-DG effectively blocked glycolysis in CSPCs with IL-1β and *iv*-hEVs (Figure 3B), it failed to restore *iv*-hEV-induced mitochondrial damage or cellular senescence in CSPCs (Figures 4D–4G and S6A–S6C). Additionally, glycolysis inhibition did not reverse the suppressed expression of cartilage anabolic genes *col2a* and *acan*, nor did it reduce the elevated expression of catabolic genes *mmp13* and *adamts5* (Figures 4H, 4I, and S6D).

### hEVs induce mitochondrial damage of OA CSPCs in an ADT1-dependent manner

**Figure 5:**
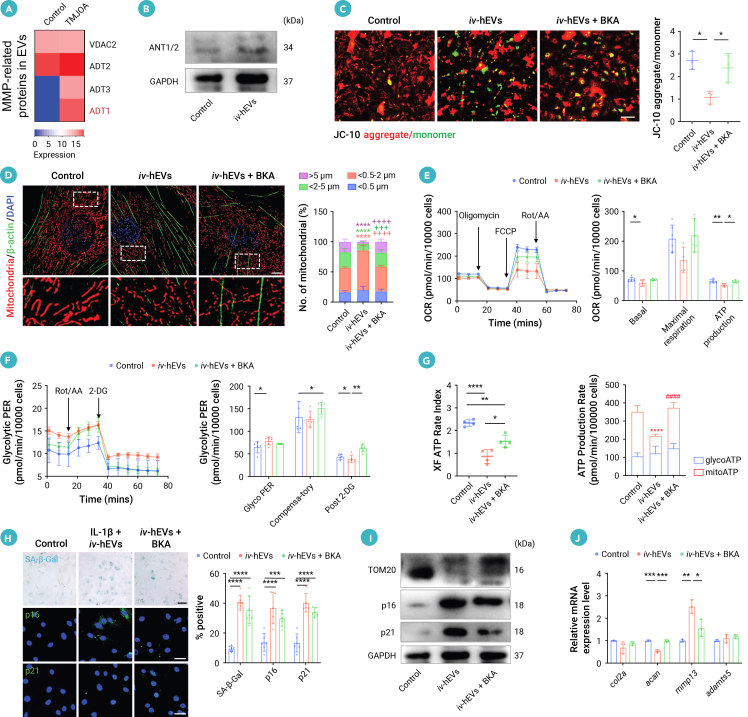
hEVs induce ADT1-dependent mitochondrial dysfunction (A) Heatmap of mitochondrial-related DEPs of joint fluid EVs from TMJOA patients versus controls. The most significantly upregulated protein related to ΔΨm, ADT1, was labeled. (B) Western blotting analysis of ANT1/2 expression in *iv*-hEV-treated CSPCs. (C) JC-10 staining quantifying ΔΨm of CSPCs treated by *iv*-hEVs with or without bongkrekic acid (BKA, an ADT1 inhibitor) (*n* = 3). Scale bar, 200 μm. (D) Representative confocal images and quantification of mitochondria fragmentation in CSPCs in BKA-treated CSPCs (*n* = 5–6). Magnified images of the boxed regions are shown in the lower panel. Scale bar, 5 μm. (E–G) OCR (E), glycolytic PER (F), and ATP (G) analysis in CSPCs treated by *iv*-hEVs with or without BKA (*n* = 4–6). (H) SA-β-Gal, p16, and p21 staining and quantification of senescent cells in BKA-treated CSPCs (*n* = 5–7). Scale bar, 100 μm. (I) Western blotting analysis of TOM20, p16, and p21 expression in BKA-treated CSPCs. (J) RT-qPCR analysis of *col2a*, *acan*, *mmp13*, and *adamts5* expression in CSPCs or incubated with *iv*-hEVs or *iv*-hEVs and BKA for 24 h (*n* = 3). Data represent mean ± SD. ∗∗∗∗*p* < 0.0001 versus control and ^+++^*p* < 0.001, ^++++^*p* < 0.0001 versus *iv*-hEVs (D); ∗*p* < 0.05, ∗∗*p* < 0.01, ∗∗∗*p* < 0.005, and ∗∗∗∗*p* < 0.0001 (C, E–H, and J) by one-way ANOVA with Tukey’s test.

The above observations inspired us to investigate the mechanisms by which *iv*-hEVs directly damage mitochondria. Given the significant changes in mitochondrial morphology observed in CSPCs exposed to *iv*-hEVs, we first examined mitochondrial fusion and fission genes through RNA-seq analysis. Surprisingly, no significant alterations were observed when comparing CSPCs treated with IL-1β and *iv*-hEVs to those treated with IL-1β alone (Figures S7A and S7B). Thus, we analyzed the mitochondrial outer membrane permeabilization-related proteins in the proteomic data of OA EVs to identify candidate proteins that might affect the ΔΨm of chondrocytes (Figure 5A). Higher levels of ADT1, ADT2, and ADT3, proteins known to negatively affect ΔΨm,^33^ were detected in OA EVs compared to control EVs. At the same time, a similar level of VDAC2, which is positively associated with ΔΨm,^34^ was observed. Among these, ADT1 displayed the most pronounced elevation (Figure 5A). Western blotting analysis further confirmed that *iv*-hEV treatment notably increased ANT1/2 protein expression in CSPCs (Figure 5B). Additionally, we performed further Western blot analysis to confirm the protein content of *iv*-hEVs. The results showed that both the EVs from OA patients and *iv*-hEVs highly expressed ANT1/2 protein compared to EVs from healthy controls (Figure S7C).

Next, we found that ADT1 inhibitor BKA^35^ effectively restored damaged mitochondria in CSPCs exposed to *iv*-hEVs, as evidenced by the recovery of ΔΨm along with the reduction in mitochondrial fragmentation (Figures 5C and 5D). Furthermore, BKA treatment effectively reversed the *iv*-hEV-induced suppression of ATP production through mitochondrial OXPHOS and partially inhibited glycolysis, as assessed by mitochondrial respiration, ATP rates, and glycolytic PER in the CSPCs exposed to *iv*-hEVs (Figures 5E–5G). This prompted us to investigate whether BKA could also mitigate the senescent cell phenotype and the impaired chondrogenic profile induced by *iv*-hEVs. Unfortunately, BKA treatment only slightly reduced the *iv*-hEV-induced increase in senescence-associated β-galactosidase (SA-β-Gal)-, p16-, and p21-positive senescent cells, which remained elevated compared to control CSPCs (Figure 5H). Consistently, western blotting analysis showed that BKA treatment notably increased the expression of TOM20 and slightly decreased the expression of p16 and p21 in CSPCs exposed to *iv*-hEVs (Figure 5I). Additionally, RT-qPCR showed that BKA treatment elevated the expression of *acan* and decreased the expression of *mmp13*, whereas it failed to rescue the altered expressions of *col2a* and *adamts5* in *iv*-hEV-treated CSPCs (Figure 5J).

### hEVs deplete TCA metabolites and drive epigenetic dysregulation of OA CSPCs

**Figure 6:**
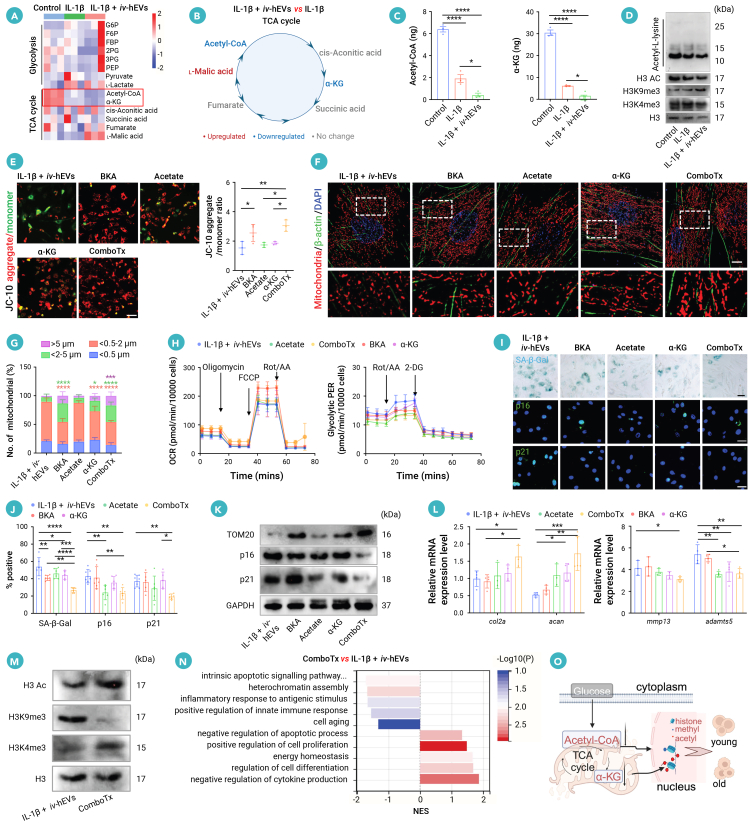
hEV-mediated TCA-cycle metabolite depletion drives epigenetic dysregulation-related chondrocyte senescence and impaired chondrogenesis (A) Heatmap of metabolite levels in CSPCs (*n* = 3), the key metabolites of the TCA cycle, acetyl-CoA and α-KG, are labeled in the heatmap. (B) Schematic diagram of how TCA-cycle-related intermediate metabolites changed in IL-1β or IL-1β + *iv*-hEV-treated CSPCs. (C) Quantification of acetyl-CoA and α-KG in CSPCs (*n* = 3). (D) Western blotting analysis of acetyl-L-lysine, acetyl-histone H3, H3K9me3, and H3K4me3 expression in CSPCs. Protein expressions were detected in CSPCs 24 h post treatment with IL-1β or IL-1β + *iv*-hEVs. (E) JC-10 staining quantifying ΔΨm of CSPCs treated alone or in combination (*n* = 3). Scale bar, 200 μm. (F and G) Representative confocal images and quantification of mitochondria fragmentation in CSPCs treated alone or simultaneously (*n* = 5–6). Magnified images of the boxed regions are shown in the lower panel. (H) OCR and glycolytic PER analysis in CSPCs of indicated groups (*n* = 5–7). (I and J) SA-β-Gal, p16, and p21 staining and quantification of senescent cells in treated CSPCs (*n* = 5–7). Scale bar, 100 μm. (K) Western blotting analysis of TOM20, p16, and p21 expression in treated CSPCs. Respective protein expressions were detected in CSPCs 24 h post treatment. (L) RT-qPCR analysis of *col2a*, *acan*, *mmp13*, and *adamts5* expression in CSPCs or treated alone and in combination for 24 h (*n* = 3–5). (M) Western blotting analysis of acetyl-histone H3, H3K9me3, and H3K4me3. Respective protein expressions were detected in CSPCs 24 h post treatment. (N) GSEA of the combination therapy versus IL-1β + *iv*-hEVs groups. (O) Schematic of the combination therapy strategy. Data represent mean ± SD. ∗*p* < 0.05, ∗∗∗*p* < 0.005, and ∗∗∗∗*p* < 0.0001 versus IL-1β + *iv*-hEVs (G); ∗*p* < 0.05, ∗∗*p* < 0.01, ∗∗∗*p* < 0.005, and ∗∗∗∗*p* < 0.0001 (C, E, J, and L) by one-way ANOVA with Tukey’s test ComboTx, combination therapy.

Mitochondrial-to-nuclear communication plays a crucial role in mediating epigenetic changes that influence aging and cell fate.^36^ To elucidate the impact of hEV-induced mitochondrial damage in CSPCs, we conducted targeted energy metabolomics profiling, focusing on glycolytic and TCA-cycle intermediates. Our results revealed that IL-1β induced elevated levels of glycolytic metabolites, specifically pyruvate and lactate in CSPCs, while simultaneously decreasing the levels of TCA-cycle metabolites, such as acetyl-CoA and α-KG (Figure 6A). Additionally, *iv*-hEV treatment intensified the changes in TCA-cycle metabolites in OA CSPCs (Figures 6B and S8A). Among these metabolites, acetyl-CoA and α-KG were notably depleted in OA CSPCs exposed to *iv*-hEVs, as confirmed by TCA-cycle metabolomic analysis and further verification through the quantification of intercellular metabolites (Figures 6A–6C).

Intracellular acetyl-CoA can directly enhance histone acetylation,^37^ while α-KG serves as a co-factor for histone demethylases that remove methyl groups from histones.^38^ These metabolic intermediates play an important role in epigenetic regulation and cell-fate determination.^39^ By analyzing differentially expressed genes (DEGs) present only in IL-1β + *iv*-hEVs versus control groups but not in the IL-1β versus control groups, GO biological process analysis indicated that *iv*-hEV treatment is involved in processes related to epigenetic modulation and cell-fate determination, including histone acetylation, maintenance of DNA methylation, heterochromatin assembly, and regulation of cartilage development (Figure S8C). To examine the changes in protein acetylation, we first performed immunoblotting specific for acetyl-L-lysine. The results revealed that *iv*-hEVs notably decreased the acetyl-L-lysine levels in the bands around 10–15 kDa, likely representing acetylated histone (H3 and H4, 10–15 kDa) acetylation, compared to IL-1β-treated and control CSPCs (Figure 6D). Additionally, the decreased level of acetyl-CoA in aging mice was associated with reduced acetylation of H3.^40^ We then found that *iv*-hEV treatment further reduced the acetylation of H3 in IL-1β-treated CSPCs (Figure 6D). Moreover, α-KG can reduce H3K9me3, which may promote stem cell differentiation.^41^ Our finding showed that *iv*-hEV treatment increased the level of the repressive histone mark H3K9me3 while reducing the level of the active histone mark H3K4me3 in IL-1β-treated CSPCs (Figure 6D), indicating a shift toward transcriptional repression in *iv*-hEV-treated CSPCs. These results indicate that mitochondrial dysfunction caused by hEVs leads to a depletion of key TCA metabolites, particularly acetyl-CoA and α-KG, driving epigenetic dysregulation associated with cellular senescence in CSPCs.

### A combination therapy combining ADT-1 inhibitor, acetate, and α-KG restores hEV-induced mitochondrial damage and senescence in OA CSPCs

Given that both irreversible mitochondrial damage and the TCA metabolite-mediated epigenetic dysregulation contribute to hEV-induced CSPC impairment, we developed a combined strategy utilizing BKA to alleviate mitochondrial damage while simultaneously supplementing with acetate (for acetyl-CoA replenishment) and α-KG to correct the epigenetic alterations. As anticipated, compared to the notably therapeutic effect of BKA on mitochondria damage in OA CSPCs exposed to *iv*-hEVs, individual administration of acetate or α-KG exhibited limited or negligible effects on the decreased ΔΨm, increased mitochondria fragmentation, and the metabolic switch from OXPHOS toward glycolysis (Figures 6E–6H and S8D–S8F). Conversely, acetate or α-KG alone proved to be more effective than BKA in reducing cellular senescence (Figures 6I–6K). Importantly, the combination therapy demonstrated superior efficacy to any individual treatments in reversing mitochondrial damage and alleviating cellular senescence in IL-1β + *iv*-hEV-treated CSPCs (Figures 6E–6K). Consequently, RT-qPCR analysis showed that combination therapy effectively elevated the expression of cartilage anabolic genes *col2a* and *acan* while notably reducing the expression of the catabolic gene *adamts5* (Figure 6L). Additionally, the combination therapy restored dysregulated histone acetylation and methylation in IL-1β + *iv*-hEV-treated CSPCs, as evidenced by increased H3 acetylation, decreased H3K9me3, and increased H3K4me3 levels (Figure 6M). Gene set enrichment analysis (GSEA) of the RNA-seq data illustrated that combination-treated CSPCs displayed enhanced transcriptional activities related to cell differentiation, energy homeostasis, and negative regulation of cytokines and apoptosis, whereas they repressed transcriptional activities in gene sets associated with heterochromatin, aging, inflammation, and apoptosis (Figure 6N). In summary, the combination therapy spontaneously restored both mitochondrial damage and epigenetic dysregulation, thereby mitigating cellular senescence and rescuing the repressed transcriptional program in OA CSPCs (Figure 6O).

### Combination therapy ameliorates OA in rat models

**Figure 7:**
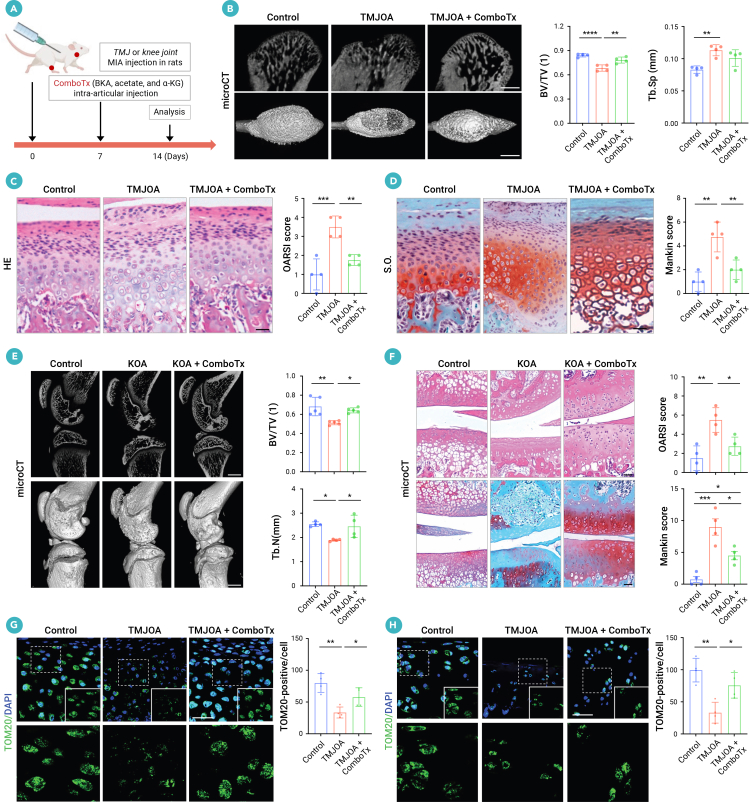
Combination therapy ameliorates OA progression in rats (A) Scheme illustrating combination treatment (BKA + acetate + α-KG) intra-articular injection procedure of TMJOA/KOA rat models. (B) Micro-CT images and quantitative analysis of subchondral bone parameters in the condylar sagittal plane (*n* = 4). Scale bar, 1 mm. (C and D) H&E (C) and SO (D) staining and histopathological scores (OARSI and Mankin) for condylar cartilage (*n* = 4). Scale bar, 200 μm. (E) Micro-CT images and subchondral bone quantification of knee joints (*n* = 4). Scale bar, 1 mm. (F) H&E and SO staining and histopathological scores (OARSI and Mankin) for knee cartilage (*n* = 4). Scale bar, 200 μm. (G and H) Immunofluorescence staining and quantification of chondrocyte mitochondrial TOM20 in TMJOA and KOA rats (*n* = 4). Scale bar, 10 μm. Data represent mean ± SD. ∗*p* < 0.05, ∗∗*p* < 0.01, ∗∗∗*p* < 0.005, and ∗∗∗∗*p* < 0.0001 (B–H) by one-way ANOVA with Tukey’s test. ComboTx, combination therapy. Please refer to Figure S9 for references.

Next, we investigated the therapeutic potential of the combination treatment for cartilage damage in OA rats (Figure 7A). Micro-CT analysis showed that the combination therapy restored the resorption of damaged condylar bone in TMJOA rats (Figure 7B). Moreover, the combination administration markedly reduced cartilage damage, as indicated by decreased hypertrophic chondrocytes and abnormal cartilage differentiation as well as enhanced proteoglycan density and glycosaminoglycan content in TMJOA rats (Figures 7C and 7D). Similarly, combination administration notably improved OA progression and cartilage damage in KOA rats, as evidenced by recovered subchondral bone loss, reduced cartilage degradation, and enhanced levels of proteoglycan and glycosaminoglycan (Figures 7E and 7F). Furthermore, TOM20 staining showed that the combination treatment rescued the mitochondrial damage and decreased the levels of senescent cells within the proliferative layer of cartilage in TMJOA and KOA rats (Figures 7G, 7H, and S9A). Furthermore, we investigated the therapeutic potential of the combination treatment for cartilage damage in OA rats injected with hEVs^high^. Compared with the aggravating effect of hEVs^high^ on OA progression, the combination treatment effectively restored hEV-exacerbated cartilage damage, as assessed by micro-CT, H&E, and SO analysis (Figures S9B–S9H). In addition, the combination treatment rescued mitochondrial damage and reduced the levels of p16- and p21-positive senescent cells in the proliferative layer of cartilage in OA rats aggravated by hEVs^high^ (Figures S9I–S9L). Taken together, these findings link hEV-mediated irreversible mitochondrial damage to chondrocyte senescence and highlight the promising potential of combination therapy for OA.

## Discussion

Metabolic homeostasis dysregulation and inflammatory stress represent central challenges in managing degenerative diseases such as OA, while conventional therapies fail to rectify metabolic imbalances and cellular dysfunction in chondrocytes or restore the compromised regenerative capacity of OA cartilage.^7^ EVs are recognized as critical mediators of intercellular communication and metabolic regulation, but their pathogenic role in driving cartilage degeneration remains poorly understood. This study revealed that humoral collagen II^+^CD86^+^ hEVs in the joint fluid are novel orchestrators of OA progression through a unique metabolic-epigenetic axis. We provide new evidence that hEVs induce irreversible mitochondrial dysfunction in OA chondrocytes, deplete TCA-cycle metabolites (acetyl-CoA and α-KG), and reprogram CSPC fate via dysregulated histone modifications. These findings uncover the essential role of EVs in regulating the metabolic balance of OA CSPCs and promote our understanding of the potential implications for broader degenerative conditions mediated by EVs.

EVs are universally present in the human body and are critical in regulating various biological processes.^42^ They facilitate the transport of bioactive proteins, mRNAs, miRNAs, and other small molecules to adjacent cells or distant organs.^12^ These features enable EVs to be potent natural-born regulators in a variety of diseases. Previous studies on the pathology of OA have largely focused on individual cell types or tissue-derived extracellular derivatives detected in joint fluid, such as EVs, extracellular matrix, and inflammatory factors^43^^,^^44^ while often neglecting the biological diversity and complexity of endogenous extracellular derivatives within this environment. In this study, we identified a distinct subpopulation of hEVs in joint fluid from OA patients, characterized by dual surface markers of proinflammatory macrophages and chondrocytes. The abundance of these hEVs strongly correlates with disease severity. They facilitate crosstalk between macrophages and chondrocytes, intensifying inflammation-driven glycolytic reprogramming and leading to irreversible mitochondrial fragmentation in OA CSPCs. This cascade results in diminished chondrogenic capacity and accelerated cellular senescence. The fusion of EVs with target cell membranes requires the involvement of SNARE-complex and syntaxin binding protein 1.^45^ Additionally, the expression of canonical adhesion molecules on the EV surface, including integrins, tetraspanins, and lactoferrin, contributes to cell-EV adhesion.^46^ These molecules may also contribute to the adhesion and fusion of EVs with one another, offering critical insights into the potential biological mechanisms underpinning the formation of hEVs in joint fluid. Importantly, in addition to OA, other degenerative diseases also manifest signs of mitochondrial damage, metabolic disorders, and accelerated cellular aging.^47^^,^^48^ Therefore, it is imperative to explore a broader spectrum of the characteristics and functional mechanisms of hEVs in other degenerative conditions. In future studies, it will be important to directly target the formation of these hEVs as well as using gene-editing strategies targeting chondrocytes and macrophages to modulate hEV formation to treat cartilage damage in OA.

Current understanding of the OA environment reveals that chondrocytes undergo pathological changes in metabolic homeostasis and cartilage remodeling. Previous studies have demonstrated specific patterns of altered chondrocyte metabolism in OA. However, the pathways through which endogenous stressors influence these metabolic alterations, as well as changes related to key regulatory enzymes of glycolysis and intermediates of the TCA cycle, remain largely unclear. In this study, treatment with 2-DG, a glycolysis inhibitor, failed to correct the metabolic imbalance or chondrocyte dysfunction induced by hEVs. Insights from recent investigations in osteoblasts indicate that while maintaining a minimal ATP threshold is essential for cellular function, the effects of mitochondrial morphological changes during osteoblast maturation are more influential than minor fluctuations in ATP generation. This highlights the significant physiological and pathological implications of mitochondrial morphological dynamics in cellular processes.^49^ Importantly, our findings suggest that mitochondrial morphological changes are not the primary cause of irreversible metabolic abnormalities in this study. Instead, hEVs inhibit the ΔΨm in chondrocytes in an ADT1-dependent manner, leading to irreversible mitochondrial damage and metabolic dysfunction, which is crucial for the fate regulation of CSPCs. Consistent with previous reports on the role of ADT1 in mitochondrial function regulation,^50^ our study found that ADT1-mediated changes in ΔΨm are crucial for its metabolic regulatory effects. Previous studies showed that IL-1β induces significant mitochondrial dysfunction in chondrocytes, which can be partially restored by adenosine treatment.^51^^,^^52^ Additionally, the activation of adenosine A2A receptor diminished senescence of chondrocytes *in vitro* and in OA models by regulating p53.^53^ These findings are consistent with our observations that IL-1β-induced mitochondrial injury and CSPC senescence can be reversed upon the removal of inflammatory stimuli. In this study, we further demonstrated that hEVs lead to irreversible mitochondrial injury and sustained cellular senescence in IL-1β-treated CSPCs.

Mitochondria-nucleus crosstalk, particularly the mitochondrial metabolite-epigenetic axis, enables cells to adapt to metabolic changes and age-related stresses.^21^ Studies indicate that acetyl-CoA levels decline in the aging mouse brain.^40^ Our study found that hEVs significantly reduced acetyl-CoA in CSPCs, affecting histone acetylation and the transcriptional program, which was consistent with previous studies suggesting that acetyl-CoA plays a crucial role in determining cell fate and function.^54^^,^^55^ Furthermore, α-KG is an important metabolic intermediate for epigenetic enzymes that regulate histone demethylation and transcriptional reprogramming.^56^ Previous studies emphasize that low levels of H3K9me3 are vital for maintaining embryonic stem cell pluripotency.^41^ Conversely, aging has been linked to an increase in H3K9me3 (associated with gene repression) positive cells in bone marrow,^57^ along with a decrease in H3K4me3 (associated with gene activation) levels in hematopoietic stem cells.^58^ In our study, hEV treatment further enhanced the expression of H3K9me3 in OA CSPCs while decreasing H3K4me3 expression. Future investigations should explore the specific roles of acetyl-CoA and α-KG in determining cell fate in the epigenetic landscapes.

Growing evidence highlights the pathogenic heterogeneity of OA, with emerging molecular mechanisms elucidating its clinical diversity. Our findings reveal OA as a multifactorial disease involving not only metabolic dysregulation but also intricate epigenetic changes. This further underscores the limitations of current “one-fits-all” treatment. By integrating the benefits of the mitochondrial protectant with metabolite supplementation, we have developed a combination therapy to address chondrogenesis disorders. This approach has demonstrated significant therapeutic effects in TMJOA and KOA rat models. This trifecta of therapeutic outcomes surpasses what isolated strategies can achieve. Furthermore, our work provides a valuable framework for addressing degenerative diseases characterized by mitochondrial dysfunction and epigenetic dysregulation. However, potential adverse effects of this combination therapy require careful consideration. BKA may reduce ATP availability in stressed articular cartilage, impairing matrix synthesis by blocking the mitochondrial permeability transition pore.^35^^,^^59^ Additionally, excessive acetyl-CoA can lead to lipid accumulation in chondrocytes, possibly contributing to OA.^60^^,^^61^ Acetyl-CoA and α-KG supplementation may also act as nutrient sensors, linking metabolic disruption to epigenetic changes in OA.^62^^,^^63^ Therefore, careful intra-articular delivery and dose optimization are crucial to minimizing risks while preserving therapeutic benefits. Prior work has indicated that adenosine signaling through the A2A receptor (A2AR) can partially reverse these deleterious effects and diminish the senescence of chondrocytes *in vitro* and *in vivo* by reducing wild-type p53 and increasing p53 alternative splicing concomitantly, especially in A2AR knockout-induced and obesity-induced OA models.^53^ Combining A2AR activation with our combination therapy may provide synergistic benefits, particularly in advanced OA, where both senescence and metabolic dysfunction co-exist. In future experiments, it will also be important to determine the mechanism underlying the formation of these hEVs as well as the potential contributions of other EV subtypes to OA. A deeper understanding can pave the way for gene-editing strategies targeting chondrocytes and macrophages to modulate hEVs. Our *in vitro* findings demonstrated the functional effects of hEVs on chondrocytes compared to single-source EVs. These results suggest that hEVs not only aggravate but also directly lead to the damage to CSPCs, which may have a detrimental effect on the onset of OA pathogenesis. However, future studies are needed to clarify the potential role of hEVs as a pathogenic factor in OA and the underlying mechanisms. Additionally, while we examined the combination therapy in rat models, these findings may not accurately reflect the therapeutic effects in human patients, highlighting the need for validation in future translational studies.

## Resource availability

### Materials availability

This study did not generate new unique materials/reagents.

### Data and code availability

All datasets utilized or generated in this study are available from the corresponding author upon reasonable request.

## Funding and acknowledgements

This work was supported by grants from the 10.13039/501100001809National Natural Science Foundation of China (82170924 to X.K. and 82370985 to Q.Z.), Guangdong S&T Program (2025A04J7156 to X.K.), and the 10.13039/100016691Pearl River Talent Recruitment Program (2019ZT08Y485 to X.K.).

## Author contributions

T.X. and R.Z. contributed equally to designing the study plan, performing the experimental procedures, and drafting the manuscript; Xuanyi Li, Xin Li, J.W., J.L., and Y.L. contributed to performing the experiments and data acquisition, analysis, and interpretation; R.Z., Q.Z., C.Z., and S.Z. were responsible for the clinical samples; and L.C., Q.Z., and X.K. contributed to experimental design, manuscript writing, and supervision. All authors approved the final version of the manuscript.

## Declaration of interests

X.K. and T.X. are inventors on a patent application (202511241895.2) for diagnostic reagents and therapeutic drugs for OA, as described in this study.

## Supplemental information

Document S1. Figures S1–S9, Tables S1–S3, and Supplemental materials and methods

Document S2. Article plus supplemental information
