# Supplementary material for: Hybrid extracellular vesicles drive irreversible mitochondria damage and TCA metabolite deficiency-related chondrocyte senescence
Source: Innovation (Camb). 2026 Jan 5;7(4):101247. doi: 10.1016/j.xinn.2025.101247 (PMC13069418; doi:10.1016/j.xinn.2025.101247)
Supplement: Document S1. Figures S1–S9, Tables S1–S3, and Supplemental materials and methods [file mmc1.pdf]

The Innovation, Volume 7

Supplemental Information

Hybrid extracellular vesicles drive irreversible mitochondria damage and TCA metabolite deficiency-related chondrocyte senescence

Ting Xiang, Rong Zhang, Xuanyi Li, Xin Li, Jinyang Wang, Jiaqi Li, Yongxi Lu, Chi Zhang, Shangbin Zhang, Lili Chen, Qingbin Zhang, and Xiaoxing Kou

Supplemental Information

Supplemental methods and materials

Animals

OA animal models were established as described previously.[1,2] TMJOA was induced by injection of 0.5mg monosodium iodoacetate (MIA) in 50 μl saline (Sigma, Saint Louis, USA), while KOA was induced by administering 3 mg MIA in 50 μl saline. The control group was injected 50 μL sterile saline. For the intra-articular injection of EVs derived from OA joints, we isolated joint EVs from the TMJ of control and OA rats. The proportion of collagen $II^{+}CD86^{+}$ hEVs was calculated. EVs derived from OA joints, which contained a higher proportion of hEVs, were classified as the hEV^{high} group. EVs from healthy joints, which had a lower proportion of hEVs, were classified as hEV^{low} group. One week after OA induction, the rats received an intra-articular injection of joint EVs (1.5×10^9 particles in 50 μL sterile saline per rat) or an equal volume of vehicle. The proportions of hEVs for each experiment were indicated in relative figure legends.

For joint EVs isolation, the joint tissues from another batch of OA and control rats were minced with scissors and digested with 2 mg/ml type II collagenase (Sigma, C2-BIOC) in a serum-free medium. The digestion was performed at 37 °C for 60 minutes with agitation, followed by centrifugation at 800g for 10 minutes and 2000g for 10 minutes at 4 °C to remove cell debris. Next, the supernatant was centrifuged at 120000g for 120 minutes at 4 °C to isolate EVs and then washed once with 0.1 μm-filtered PBS.

Primary cell cultures

The isolation and culture of CSPCs were performed as reported.[3,4] CSPCs at the first-third passages were used for all experiments. A total of 5×10^5 cells were seeded on 6 cm culture dishes and cultured at 37 °C in 5% CO_2 with complete medium (DMEM/F12 supplemented with 10% FBS and 1% penicillin/streptomycin). After reaching 90% confluence, CSPCs were treated with 10 ng/mL IL-1β (PeproTech, 200-01B), either alone or in combination with *iv*-hEVs (2.5×10^8 particles/mL) for 24 hours. Bone marrow-derived macrophages (BMDMs) were obtained from SD rats to induce macrophage differentiation as described.[5] For M1 polarization, BMDMs were

incubated with 20 ng/mL recombinant INF-γ (PeproTech, 315-05) plus 100 ng/ml lipopolysaccharide (Sigma, 8360) for 24-48 hours.

Nanoparticle tracking analysis (NTA)

Joint fluid EVs were diluted in 0.1 μm-filtered PBS. Then, the average particle size and electric potential were measured with a ZetaView PMX120 (Particle Metrix, Germany).

Transmission Electron Microscopy (TEM)

For TEM, a drop of EV suspension was applied to carbon-coated 200 mesh grids for 5 minutes, washed with PBS, and stained with 2% uranyl acetate for 30 seconds. For immunogold staining, EVs were incubated with an equal volume of 2% glutaraldehyde for 20 minutes and applied to carbon-coated 200 mesh grids. Following blocking with 5% BSA (Servicebio, GC305010), the grids were exposed to a 1:20 dilution of primary antibodies (anti-CD86 and anti-collagen II) for 1 hour, washed with PBS, and treated with 10-nm gold-labeled (Solarbio, K1032G-G10) and 35-nm gold-labeled secondary antibodies (Solarbio, K1034G-G35) for 30 minutes. The grids were then stained with 2% uranyl acetate for 30 seconds. Images were obtained using a TEM.

Nano-flow cytometry analysis

For nano-flow cytometry analysis of EVs, joint fluid EVs, joint EVs or *iv*-hEVs were harvested. The samples were analyzed using nano-flow cytometry according to the reported method.[6] For the negative control, joint fluid EVs were resuspended in blank buffer without any staining. PE anti-human CD9 (1:50, 312105, Biolegand), FITC anti-human CD3, B220, CD11b, CD68 antibody (1:50, 344803, 103205, 982614, 333805, Biolegand), PE anti-human CD86 (1:50, 374205, Biolegand) antibodies were used. The mixture was incubated at 4°C for 30-60 minutes, and unbound antibodies were then washed once with filtered PBS by ultracentrifugation at 120,000g for 120 minutes at 4°C. Regarding the nano-flow cytometry analysis of EVs for collagen II. Following primary antibody (1:100, ab34712, Abcam) staining at 4 °C for 30-60 minutes, we stained the samples with Alexa Fluor® 488-conjugated secondary antibody (1:50, A12379, ThermoFisher), then performed an additional wash step using filtered PBS through ultracentrifugation (120,000g, 120 minutes, 4°C). After washing, EVs were resuspended in filtered PBS, diluted to the corresponding concentration range, and

tested on the Flow Nano Analyzer (Nano FCM Inc, Fujian, China). Double staining of EVs for nano-flow cytometry analysis was conducted as reported.[6,7] Briefly, two single-photon counting avalanche photodiodes (APDs) were used for the simultaneous detection of side scatter (FF01-488/6) and green FL (FF01-525/45) of individual particles/EVs, respectively.

Flow cytometric analysis

CSPCs were stained with anti-CD45-Pacific Blue (1:100, 103126, Biolegand), anti-CD90-PE (1:100, 554898, BD Biosciences), anti-CD44-PE (1:100, 553134, BD Biosciences), anti-CD11b-APC-Cy7 (1:100, 101226, Biolegand), anti-CD29-PE (1:100, 562801, BD Biosciences) at 4 °C respectively. Neutralization and centrifugation of antibodies were performed 30 minutes later, and detection was performed after resuspension with PBS by flow cytometry (NovoCyte, ACEA Biosciences). NovoExpress software was used for data analysis.

Proteomic analysis

Protein lysates of joint fluid EVs were prepared and passed to a tandem mass spectrometer Q-Exactive HF X (Thermo Fisher Scientific, San Jose, CA) for DDA (Data Dependent Acquisition) mode detection before LC-MS/MS analysis. The raw data were analyzed using HISAT2 (v2.2.1). Proteins were identified by comparing against the Uniport database with filtering performed with PSM-level FDR <= 1% and false discovery rate (FDR) set at 0.01 for both peptides and proteins. Proteins were quantified using the default parameters in MaxQuant. Proteins that were significantly upregulated in EVs were included for further functional analysis based on GO and KEGG databases. (For human samples, joint fluid EVs and protein samples were obtained from 12 patients in each group.)

Multilineage differentiation

In vitro chondrogenesis, osteogenesis, and lipogenesis were performed using the chemically defined medium. To induce osteogenesis, CSPCs (5×10^4) were cultured in a 12-well plate for 2 weeks in the osteogenic medium containing 2 mM β-glycerophosphate (Sigma), 100 μM L-ascorbicacid 2-phosphate (Sigma) and 10 nM dexamethasone (Sigma). Calcium nodules were visualized by staining with alizarin

(Sigma). For adipogenesis, CSPCs (5 x 10^4) were cultured in a 12-well plate for 2 weeks under adipogenic inductive conditions in growth medium containing 500 nM isobutylmethylxanthine (Sigma-Aldrich), 60 µM indomethacin (Sigma-Aldrich), 500 nM hydrocortisone (Sigma-Aldrich), 10 µg/mL insulin (Sigma-Aldrich), and 100 nM l-ascorbic acid phosphate. Adipogenic differentiation capabilities were analyzed using oil red O staining (Sigma-Aldrich). For chondrogenesis, CSPCs (2.5 x 10^5) were cultured in a 6-well plate in the chondrogenic induction medium supplemented with 5% FBS (ExCell Bio), 1% P/S (Invitrogen), 1% ITS-G (ThermoFisher) and 50 µM ascorbic acid (Sigma). The culture was maintained for 7 days, followed by Alcian blue (Solarbio) staining for observation.

Mitochondrial morphology observation

MitoTracker™ Red, a Δψm-sensitive fluorescent dye, was employed for mitochondrial monitoring. When cells reached the desired confluency, cells were labeled with MitoTracker™ Red (100 nM, Invitrogen) to visualize mitochondrial morphology, and incubated at 37 °C for 30 minutes. After staining was complete, the cells were washed with fresh growth medium, and the medium was replaced with 4% paraformaldehyde (PFA). After fixation, the cells were washed several times with filtered PBS. Then, the fixed cells were incubated in PBS containing 0.5% Triton X-100 (Sigma) for 15 minutes. For immunofluorescence staining of cytoskeleton markers, cells were incubated with Alexa Fluor 488-conjugated actin for 20 minutes at room temperature. Finally, stained slides were collected with 4,6-diamidino-2-phenylindole dihydrochloride (DAPI, Beyotime) drops. The mitochondrial images were captured using the Elyra 7 Lattice SIM (Zeiss, Germany) and analyzed with Zeiss Zen Blue edition software. The measurements were further processed using Fiji software (1.54f) to calculate mitochondrial length as reported.[8,9] At least over fifty mitochondria were measured and analyzed per sample to obtain data.

Mitochondrial membrane potential (ΔΨm)

ΔΨm was measured following the manufacturer's protocol (Yeasen). JC-10 fluorescence images were captured using an inverted fluorescence microscope. The ratio of fluorescence intensity between JC-10 aggregates and monomers was calculated

to assess alterations in $\Delta\Psi m$.

SA-β-gal staining

The cellular senescence assays of frozen sections and CSPCs (5 x 10^4 cells/group) were performed following the instructions provided by the Cellular Senescence Assay Kit (Solarbio).

Histological staining and micro-computed tomography (Micro-CT) scanning

After being harvested and fixed in 4% PFA, TMJs and femurs were imaged and analyzed using a high-resolution Scanco μCT35 scanne (Scanco Medical AG, Switzerland). Data were visualized and analyzed using Amira 5.3.1 software.

For histological staining, tissues were removed and fixed in 4% PFA for 24 hours, followed by decalcification with 10% EDTA (pH 7.4, BioFRoxx). Subsequently, the paraffin-embedded sections (4 μm) were prepared for HE, SO, and TB staining. The histomorphology grade was evaluated using a modified assessment system for OA grade based on the OARSI and Mankin scoring systems.

Biodistribution of EVs

EVs were labeled with PKH26 (Sigma) and then injected into the joint cavity of OA rats. The rats were killed at different time points of 2, 4, and 6 hours after injection. The condylar tissue of the joint was taken and imaged by the IVIS *in vivo* imaging system.

Immunofluorescent staining

Paraffin sections were routinely dewaxed with xylene and alcohol, antigen were repaired with sodium citrate, and then treated with 3% hydrogen peroxide (H_2O_2) for 15 minutes. After thorough washing with PBS, the sections were blocked with 5% BSA for 60 minutes and then incubated with primary antibodies overnight at 4°C. The primary antibodies used were as follows: anti-TOM20 (1:200, 11802-1-AP, Proteintech), anti-p16 (1:200, R23896, Zen BioScience), anti-p21 (1:200, R381102, Zen BioScience). After primary antibody incubation, sections were washed for 3 times with PBS and incubated with appropriate Alexa Fluor-conjugated secondary antibodies for 60 minutes at room temperature. Then, sections were washed 3 times with PBS and nuclei were counterstained with DAPI. Photographs were taken by a confocal microscope (LSM 980, Zeiss) and analyzed using the ImageJ software (1.54j). To

investigate the *in vivo* uptake of EVs, cryosections of tissues were washed thoroughly with PBS, blocked in 5% BSA for 60 minutes, and probed with the primary antibody anti-collagen II overnight at 4 °C. The subsequent steps were identical to those outlined previously. For *in vitro* experiments, CSPCs were cultured with PKH26-prelabeled M1-macrophage-EVs and PKH67-prelabeled chondrocyte-EVs for 3 hours (Sigma). Then, samples were fixed by 4% PFA, blocked with 5% BSA, and counterstained with cytoskeletal dye for 30 minutes at room temperature. The cells were washed for 3 times with PBS and then mounted with DAPI. Photographs were taken by Elyra 7 Lattice SIM (Zeiss, Germany) and analyzed using the ImageJ software (1.54j). Coloc2 was used to determine the correlation of expression levels of different fluorophore signals. For immunofluorescence staining of cell senescence markers, 5 x 10^4 CSPCs were fixed by 4% PFA, blocked with 5% BSA for 60 minutes at room temperature, and washed 3 times with PBS, then incubated with p16, p21 primary antibodies overnight at 4 °C. After primary antibody incubation, the cells were washed for 3 times with PBS, then incubated with appropriate Alexa Fluor-conjugated secondary antibodies for 60 minutes at room temperature and mounted with DAPI. Photographs were taken by a confocal microscope (LSM 980, Zeiss) and analyzed using the ImageJ software. All analyses were performed with ImageJ software (1.54j). Cell Counter was used to quantify the number of positive cells.

RNA isolation and qRT-PCR

Total RNA was extracted with RNA extraction kit (15596018, Invitrogen). For qRT-PCR of mRNA, the cDNA was synthesized using Prime Script RT Reagent Kit (RR037A, Takara). Then, qRT-PCR was conducted with SYBR Green Master Mix (Q711-03, Vazyme) and gene-specific primers. Quantification was performed by using β-actin as the internal control and calculating the relative expression level of each gene with the 2-ΔΔCT method. All the primer sequences were presented in Supplementary Table 3.

Western blotting analysis

Cells or purified EVs were harvested and lysed in RIPA (CW2333S, CWBIO) on ice. Protein concentrations were determined via a BCA protein assay kit (23225, Invitrogen).

After quantification using the BCA kit, 20 ug protein for each sample was loaded onto SDS-polyacrylamide gel electrophoresis (NP0321BOX, Invitrogen) and transferred to 0.2-μm PVDF membranes (ISEQ00010, Millipore). Membranes were blocked for 1 hour at room temperature in 5% non-fat dry milk and 0.1% Tween-20, followed by overnight incubation at 4°C with primary antibodies targeting CD9 (ab236630, Abcam), CD81 (sc-70803, Santa Cruz Biotechnology), CD63 (sc-5275, Santa Cruz Biotechnology), Calnexin (sc-23954, Santa Cruz Biotechnology), GPI (15171-1-AP, Proteintech), ALDOB (18065-1-AP, Proteintech), PGK1 (17811-1-AP, Proteintech), ANT1/2 (17796-1-AP, Proteintech), TOM20 (11802-1-AP, Proteintech), VDAC2 (ab154856, Abcam), p16 (R23896, Zen BioScience), p21 (R381102, Zen BioScience), GAPDH (10494-1-AP, Proteintech), Acetyl-L-lysine (HA723073, HUABIO), Acetyl-Histone H3 (Lys9/14/18/23/27, AF3359, Affinity Biosciences), H3K9me3 (ab8898, Abcam), H3K4me3 (A22146, Abclonal) and Histone H3 (06-570, Millipore). After washing with TBS containing 0.1% Tween-20, membranes were incubated with species-specific horseradish peroxidase-conjugated secondary antibodies for 1 hour at room temperature. Protein bands were visualized using SuperSignal West Pico PLUS (34580, Thermo Scientific) Chemiluminescent Substrate and SuperSignal West Femto Maximum Sensitivity Substrate (34095, Invitrogen), and detected using a ChemiDoc™ MP imaging system (Bio-Rad, USA).

Metabolomics analysis

Mitochondria were isolated using a cell mitochondria isolation kit (Beyotime), which were then analyzed for mass spectrometry detection experiment based on the LC-MS/MS platform. Specifically, energy metabolism standards were taken in the injection vial to make a standard solution for LC-MS/MS analysis. Metabolites were extracted according to the reported method.[10] The MS analysis was performed using an AB Sciex Triple Quadrupole 6500 plus mass spectrometer (AB Sciex, USA) in the multiple reaction monitoring (MRM) mode. In calculation, all metabolite concentrations less than 0 were reported as not detected (ND). Only metabolites present in > 50% of the samples were kept for further analysis.

RNA-seq analysis

Total RNA was isolated from CSPCs using Trizol according to the manual instruction. RNA sequencing libraries were generated with an insert size ranging from 100 to 500 bp, and sequenced using the BGISEQ-500 platform (BGI-Shenzhen, China). KEGG pathway enrichment analysis of CSPCs was performed using a previously reported RNA-seq dataset.[11] The expression level of gene were calculated by RSEM (v1.2.12). Data processing and analysis were performed using the OmicShare tool and BGI analysis platform.

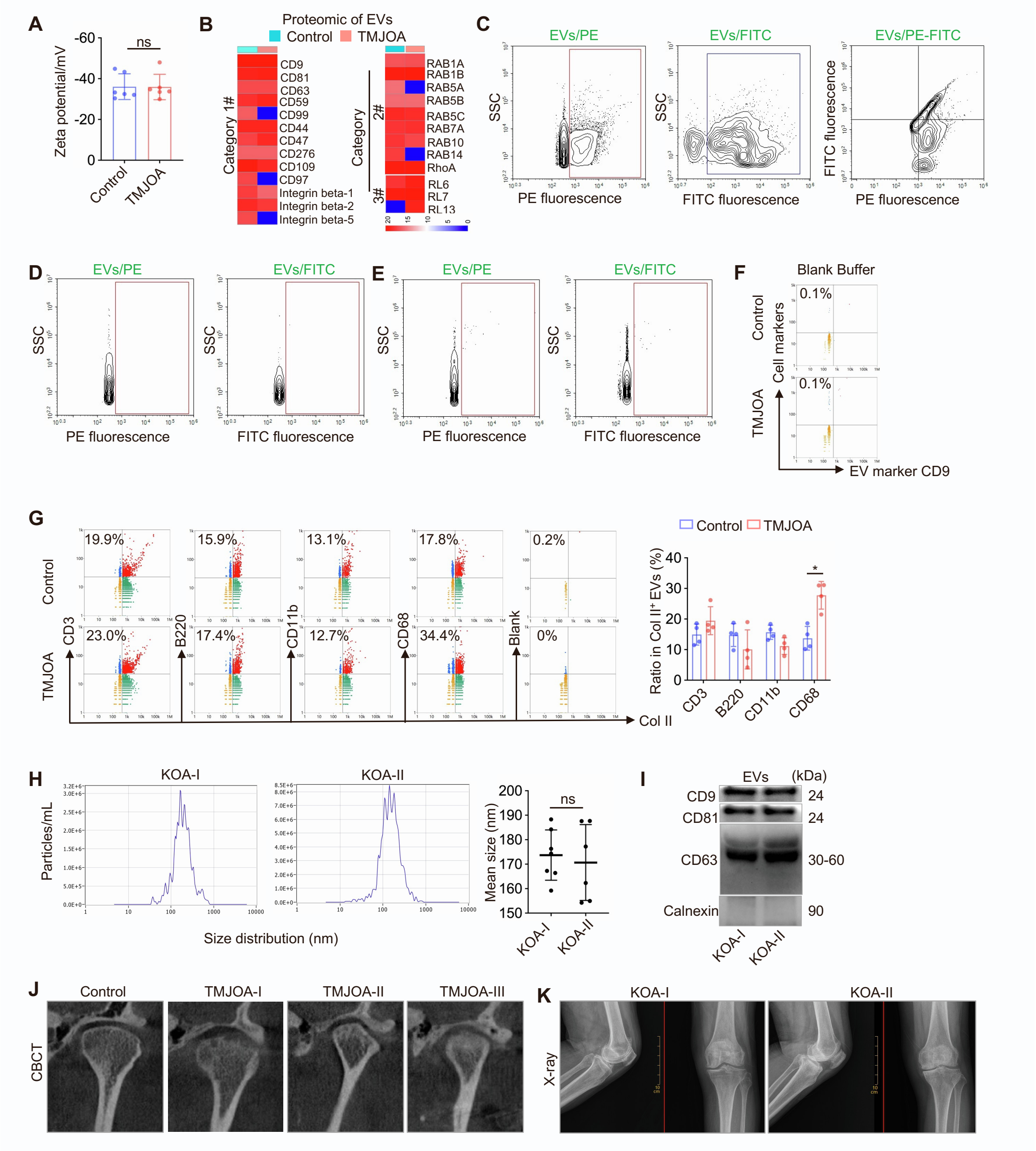

Supplementary figure 1. Characterization of hEVs in the joint fluid of OA patients. **(A)** The membrane potential of joint fluid EVs isolated from control and TMJOA patients (n = 10). **(B)** Heatmap showing DEPs in joint fluid EVs from TMJOA patients versus controls. Analysis of proteins for subtypes of EV characteristics based on the MISEV2018 guidelines. **(C-E)** Gating strategy for joint fluid EVs characterization. Joint fluid EVs were identified based on side-scattered light (SSC) and CD9 fluorescence positivity. Each EV subtype was defined using FITC-conjugated antibodies. To confirm that the recorded events could be recognized as true EVs (C). buffer-only (D). unstained treatment (E). **(F)** Negative control for nano-flow cytometry analysis in Figure 1**F**. **(G)** Nano-flow cytometry analysis showing the hybrid cellular

orientation of the EVs from joint fluid. Quantifications showing the proportions of CD3$^+$collagen II$^+$, B220$^+$collagen II$^+$, CD11b$^+$collagen II$^+$, and CD68$^+$collagen II$^+$ double positive EVs in control and TMJOA patient joint fluid (n = 4). **(H)** Size distribution of joint fluid EVs from KOA patients analyzed by NTA (n = 6-7). **(I)** Western blotting analysis of EV-positive markers (CD9, CD81, CD63) and negative marker Calnexin in EVs isolated from KOA patients. **(J and K)** Typical cone beam computed tomography (CBCT) and X-ray images of TMJOA and KOA patients. Data represent mean ± SD. ****$p < 0.0001$ by t test (**A** and **H**). *$p < 0.05$ by one-way ANOVA with Tukey's test (**G**).

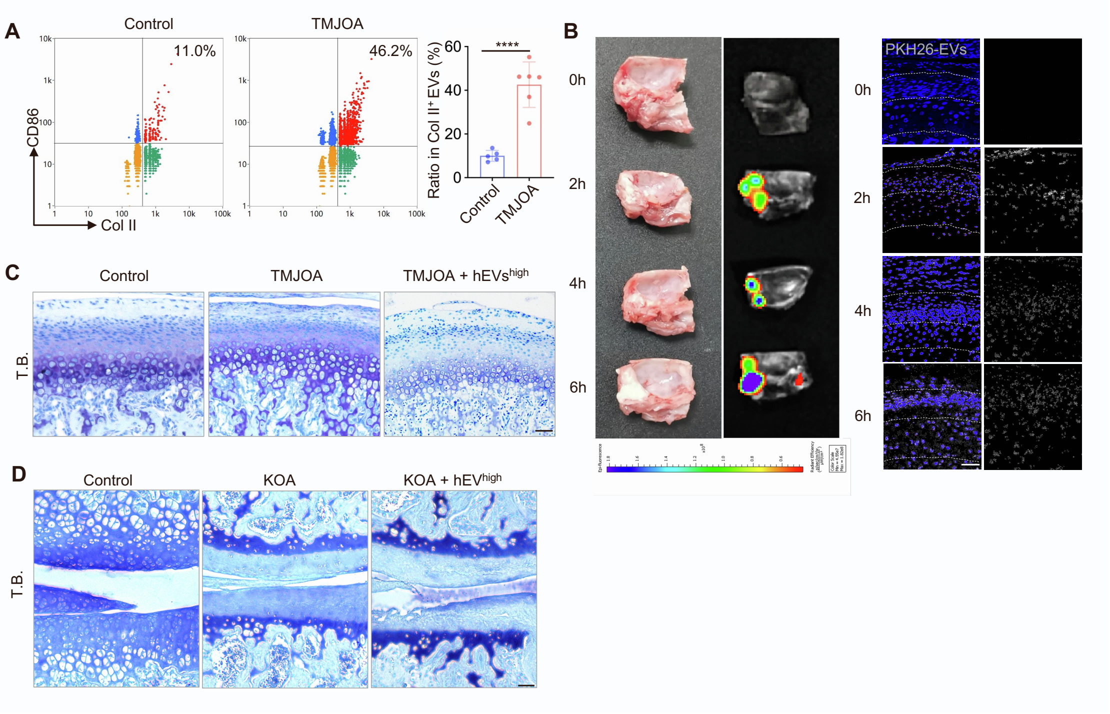

Supplementary figure 2. hEVs internalization by cartilage aggravates chondrocyte senescence in OA rats. (A) Nano-flow cytometry analysis showing the proportion of CD86$^+$collagen II$^+$ hEVs from the cartilage of TMJOA (46.2%) and control (11.0%) rats within the injected EV population (n = 5). **(B)** Representative confocal images showed time-dependent internalization of PKH26-labeled EVs (white) by chondrocytes and counterstained by DAPI (blue). Scale bar, 100 μm. **(C,D)** Toluidine blue (TB) staining of condylar cartilage and knee joints. Scale bar, 200 μm. Data represent mean ± SD. ****$p < 0.0001$ by t test (**A**).

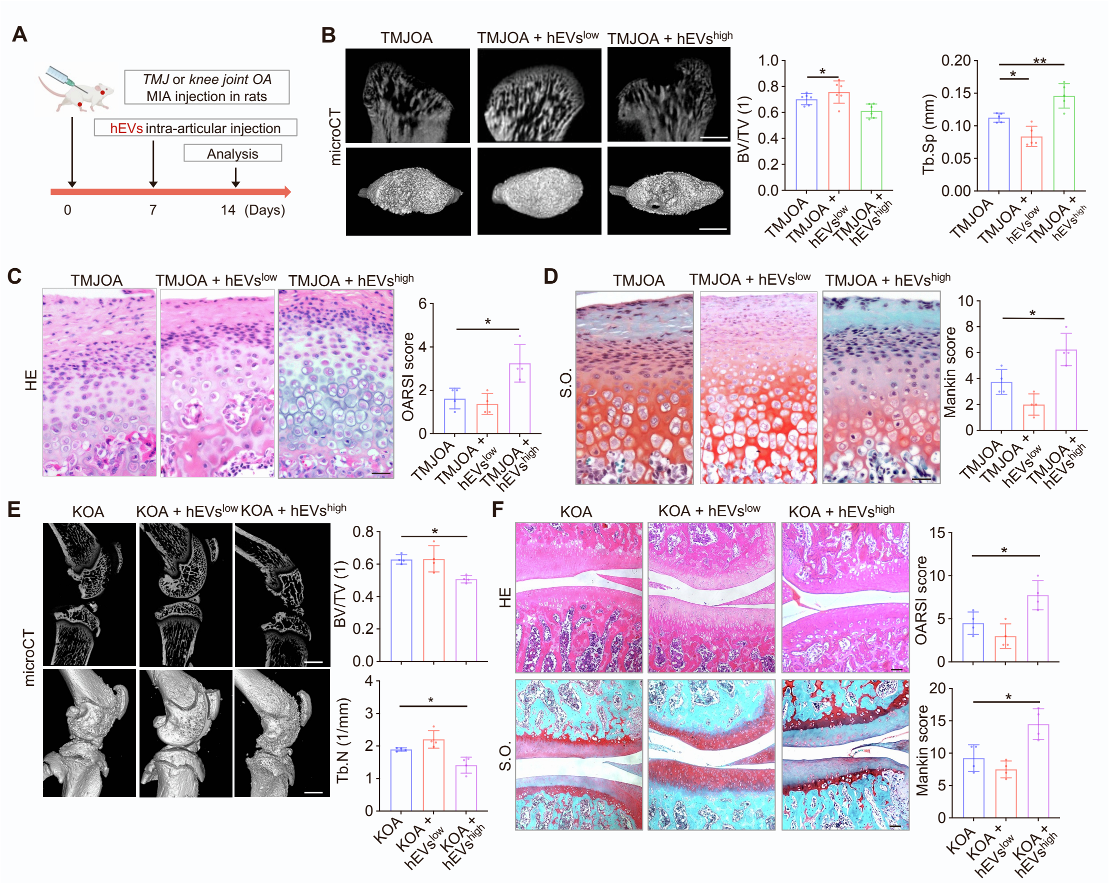

Supplementary figure 3. (A) Scheme illustrating hEV intra-articular injection procedure of TMJOA/KOA rat models. (B) Micro-CT images and quantitative analysis of subchondral bone parameters in the condylar sagittal plane (n = 5-6). Scale bar, 1 mm. (C and D) HE and SO staining and histopathological scores (OARSI, Mankin) for condylar cartilage (n = 4). Scale bar, 200 μm. (E) Micro-CT images and subchondral bone quantification of knee joints (n = 4). Scale bar, 1mm. (F) HE and SO staining and histopathological scores (OARSI, Mankin) for knee cartilage (n = 4). Scale bar, 200 μm. Data represent mean ± SD. *$p < 0.05$, **$p < 0.01$ by one-way ANOVA with Tukey's test (**B-F**).

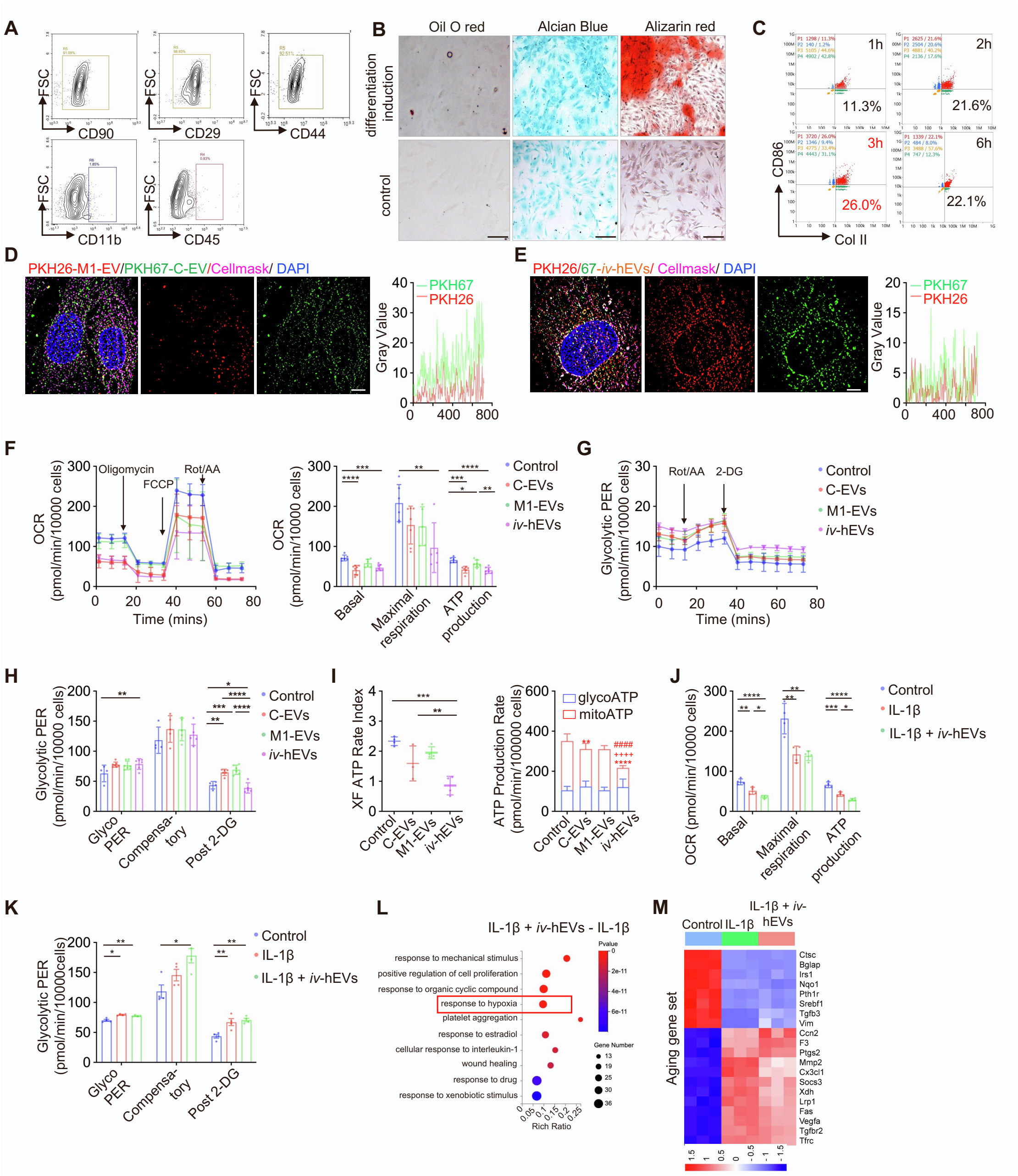

Supplementary figure 4. Identification of CSPC and hEV in vitro. (A) Flow analysis of stem cell-positive markers (CD90, CD29 and CD44) and negative markers (CD11b, CD45) in CSPCs isolated from SD rats. **(B)** Multilineage differentiation capacities of CSPCs. Scale bar, 200 μm. **(C)** Nano-flow cytometry analysis showing the fusion rate of *iv*-hEVs cultured at different time points. **(D,E)** Immunofluorescence staining and colocalization analysis of PKH26-labeled M1-EV (red), PKH67-labeled C-EV (green), and *iv*-hEVs endocytosed by CSPCs. Scale bar, 5 μm. **(F-I)** OCR, Glycolytic PER and ATP analysis in CSPCs 24 hours post-treatment of IL-1β, C-EVs,

M1-EVs, or *iv*-hEVs (n = 5-8). **(J,K)** Quantitative analysis of OCR and glycolytic PER in CSPCs of indicated groups (n = 4). **(L)** GO functional enrichment of DEGs in IL-1β + *iv*-hEV-treated CSPCs versus IL-1β treated CSPCs. **(M)** Heatmap of aging-related genes in IL-1β or IL-1β + *iv*-hEVs-treated CSPCs versus control CSPCs. Data represent mean ± SD. *$p < 0.05$, **$p < 0.01$, ***$p < 0.001$, ****$p < 0.0001$ by one-way ANOVA with Tukey's test (**F**, **H-K**).

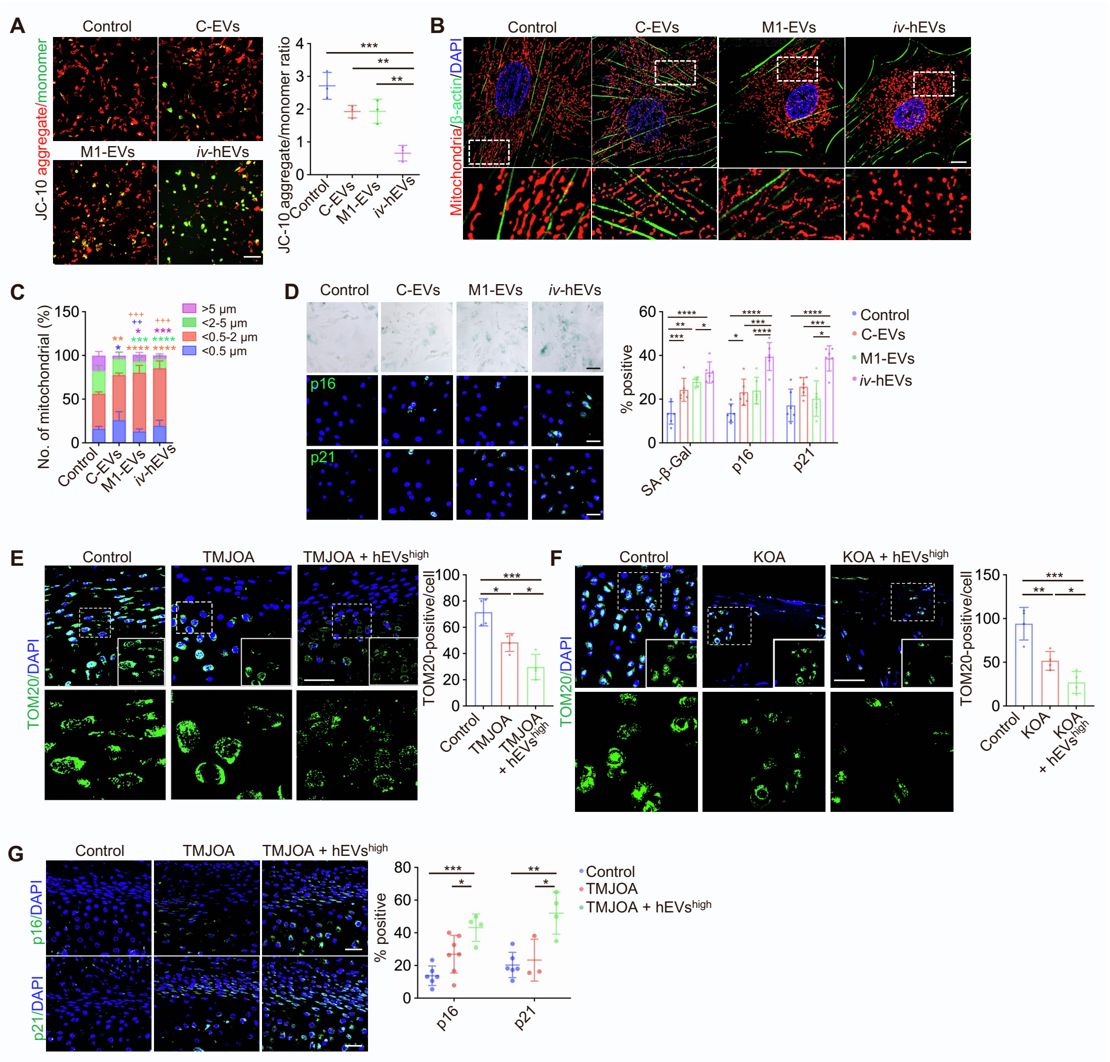

Supplementary figure 5. Enhanced destructive effects of hEVs compared to single EVs. (A) JC-10 staining quantifying MMP in CSPCs of indicated group (n = 3). Scale bar, 200 μm. **(B,C)** Representative confocal images and quantification of mitochondria fragmentation in CSPCs treated with IL-1β, C-EVs, M1-EVs, or *iv*-hEVs (n = 5-6). Magnified images of the boxed regions are shown in the lower panel. Scale bar, 5 μm. **(D)** SA-β-Gal, p16, and p21 staining and quantification of senescent cells in CSPCs treated with IL-1β, C-EVs, M1-EVs, or *iv*-hEVs (n = 5-6). Scale bar, 100 μm. **(E,F)** Immunofluorescence staining and quantification of chondrocyte mitochondrial TOM20, revealing hEV^{high}-induced mitochondrial damage in TMJOA and KOA rats (n = 4). Scale bar, 10 μm. **(G)** Representative confocal images and quantification of p16 and p21 positive chondrocyte in TMJOA rats (n = 4). Scale bar, 200 μm. Data represent mean ± SD. $*p < 0.05$, $**p < 0.01$, $***p < 0.005$, $****p < 0.0001$ versus control, $^{++}$ p

< 0.01, $^{+++}p < 0.001$ versus C-EVs (**C**); $^{*}p < 0.05$, $^{**}p < 0.01$, $^{***}p < 0.005$, $^{****}p <$ 0.0001 by one-way ANOVA with Tukey's test (**A**, **D-G**).

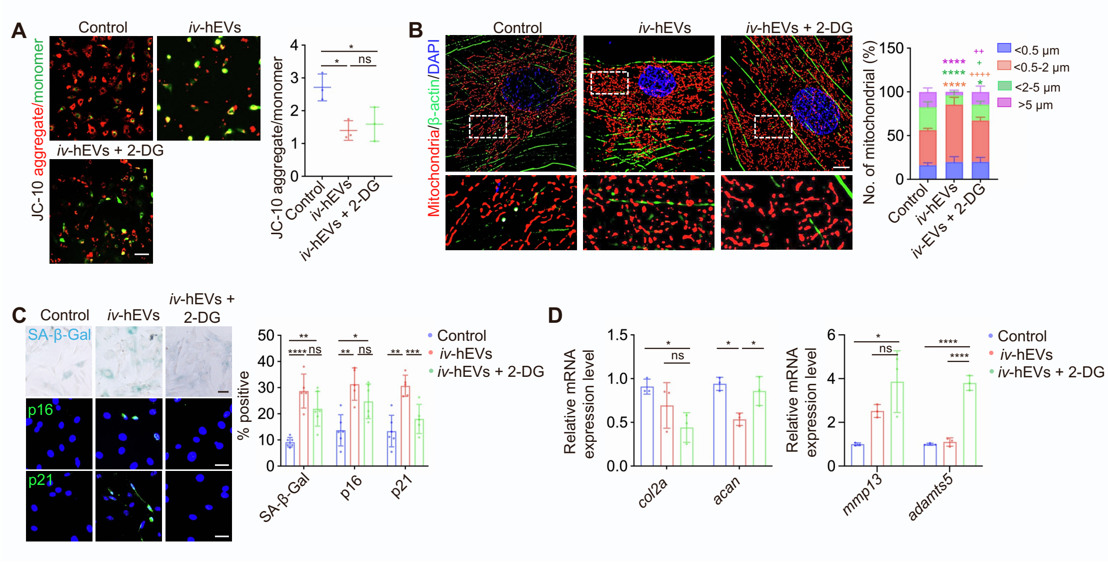

Supplementary figure 6. Glycolytic inhibition partially restores hEV-induced CSPC damage. (A) JC-10 staining quantifying MMP in CSPCs treated with *iv*-hEVs with or without 2-DG treatment (n = 3). Scale bar, 200 μm. **(B)** Representative confocal images and quantification of mitochondria fragmentation in CSPCs treated with *iv*-hEVs with or without 2-DG treatment (n = 5-6). Magnified images of the boxed regions are shown in the lower panel. Scale bar, 5 μm. **(C)** SA-β-Gal, p16, and p21 staining and quantification of senescent cells in CSPCs treated with *iv*-hEVs with or without 2-DG treatment (n = 5-6). Scale bar, 100 μm. **(D)** qRT-PCR of cartilage anabolic and catabolic genes in CSPCs treated with *iv*-hEVs with or without 2-DG treatment (n = 3). Data represent mean ± SD. $^{*}p < 0.05$, $^{****}p < 0.0001$ versus control, $^{+}p < 0.05$, $^{++}p < 0.01$, $^{++++}p < 0.0001$ versus *iv*-hEVs (**B**); $^{*}p < 0.05$, $^{**}p < 0.01$, $^{***}p < 0.005$, $^{****}p <$ 0.0001 by one-way ANOVA with Tukey's test (**A,C,D**).

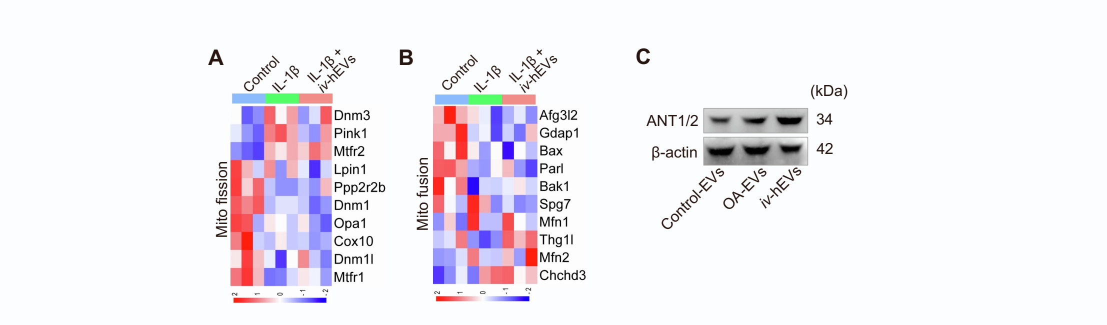

Supplementary figure 7. Mitochondrial damage induced by hEVs results in impaired membrane potential (A,B) Heatmap of mitochondrial dynamics-related genes in IL-1β or IL-1β + *iv*-hEVs-treated CSPCs. **(C)** The expression of ANT1/2 in EVs from healthy controls, OA patients and *iv*-hEVs.

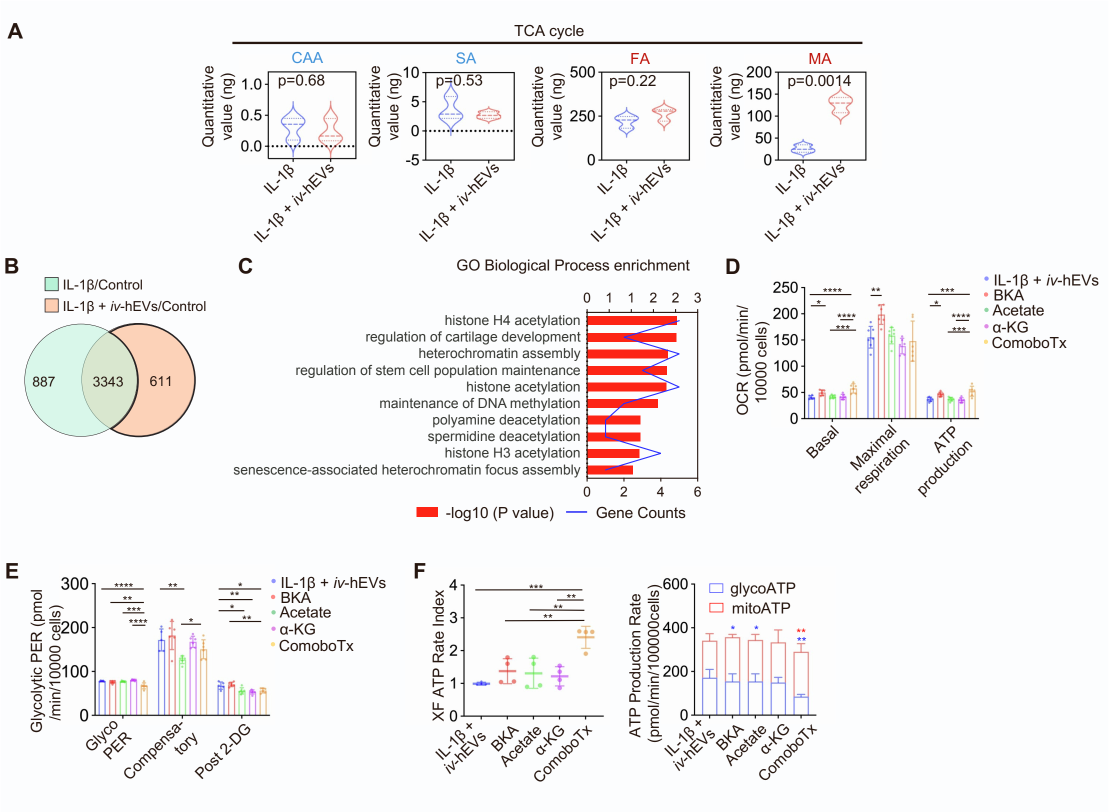

Supplementary figure 8. Combination therapy attenuates chondrocyte senescence and rejuvenates compromised chondrogenesis. (A) Violin plots of key metabolites' quantitative levels in the TCA cycle of IL-1β + *iv*-hEVs vs IL-1β-treated CSPCs. **(B)** Venn diagram of the overlap of DEGs in CSPCs. **(C)** GO functional enrichment of the overlap DEGs from the Venn diagram. **(D,E)** Quantitative analysis of OCR and glycolytic PER in CSPCs (n = 5-7). **(F)** ATP production in CSPCs of indicated groups (n = 4). Data represent mean ± SD. *$p < 0.05$, **$p < 0.01$, ***$p < 0.005$, ****$p < 0.0001$ by one-way ANOVA with Tukey's test (**D-F**).

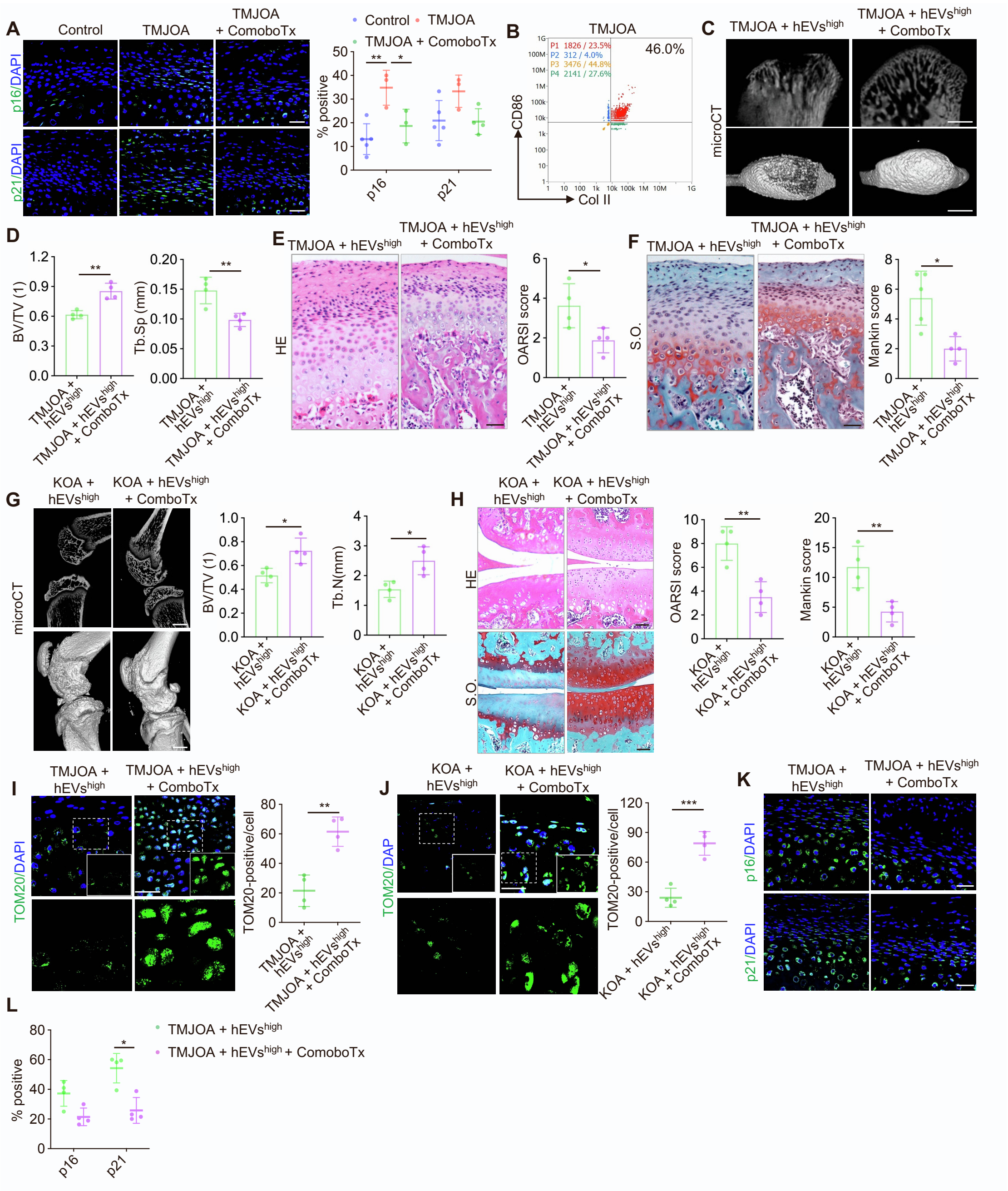

Supplementary figure 9. Combination therapy moderates chondrocyte senescence in OA rats. (A) Representative confocal images and quantification of p16 and p21 positive chondrocyte in TMJOA rats (n = 4). Scale bar, 200 μm. **(B)** Nano-flow cytometry analysis showing the proportion of $CD86^{+}$collagen II^{+} hEVs (46.0%) within the injected EV population. **(C and D)** Micro-CT images and quantitative analysis of subchondral bone parameters in the condylar sagittal plane (n = 4). Scale bar, 1 mm. **(E and F)** H&E and SO staining and histopathological scores (OARSI, Mankin) for condylar cartilage (n = 4). Scale bar, 200 μm. **(G)** Micro-CT images and subchondral

bone quantification of knee joints (n = 4). Scale bar, 1mm. **(H)** H&E staining and histopathological scores (OARSI) for knee cartilage. (n = 4). Scale bar, 200 μm. **(I and J)** Immunofluorescence staining of chondrocyte mitochondrial TOM20 in TMJOA and KOA rats (n = 4). Scale bar, 10 μm. **(K and L)** Representative confocal images and quantification of p16 and p21 positive chondrocyte in TMJOA rats (n = 4). Data represent mean ± SD. *$p < 0.05$, **$p < 0.01$, ***$p < 0.005$ by t test (**D-J**). *$p < 0.05$, **$p < 0.01$ by one-way ANOVA with Tukey's test (**A** and **L**).

Supplementary Table 1: Demographic and hEVs ratios in control and TMJOA groups						
	Age	Gender	CBCT	Pain	DI	CD86^{+} (%)
Control	25	F	normal	-	I	10.3
	35	F	normal	-	I	19.8
	28	F	normal	-	I	16.2
	43	F	normal	-	II	54
	18	F	normal	-	II	30.6
	18	F	normal	-	II	26.5
	27	F	normal	-	I	27.5
	43	M	normal	-	II	30.4
	35	F	normal	-	I	11.2
	14	F	normal	-	I	7.2
	21	F	normal	-	I	19.8
	21	F	normal	-	I	10.5
TMJOA	27	F	3	-	I	16.1
	36	F	3	+	II	51.1
	53	F	4	+	III	26.4
	53	F	4	+	III	46.5
	36	F	2	+	II	31
	18	F	7	+	II	49.6
	21	F	10	-	II	62.8
	21	F	10	-	II	24.2
	21	F	2	-	II	41.1
	21	F	2	-	II	38.7
	21	F	2	+	II	24.2
	23	F	14	+	III	38.7
	23	F	14	+	III	41.1
	23	F	14	+	III	56.7
	28	F	12	+	I	53.1
	14	F	12	+	II	51.3
	14	F	12	+	II	51.3
	14	F	12	+	II	61
	39	M	2	-	II	86
	28	F	4	+	I	53.5
	37	F	2	-	I	78.9
	15	F	4	-	I	65.9
	27	F	2	-	II	42.4
	42	F	4	+	II	60
	40	F	2	+	II	42.1
	53	F	4	-	II	50.8
	36	F	4	+	III	59.1
	27	F	9	-	III	63
	36	F	8	+	I	26.6

Supplementary Table 2: Demographic and hEVs ratios in KOA groups

	Age	Gender	CBCT	$CD86^{+}$ (%)
KOA	68	M	III	16.5
	68	F	III	9.6
	58	F	III	7.2
	70	F	IV	32.1
	78	F	III	27.8
	76	M	III	36.8
	58	F	IV	39.5
	60	F	IV	45.2
	68	M	III	20.7
	68	F	IV	74.3
	45	F	III	54.8
	52	F	IV	61.9
	70	F	III	53.3
	70	F	IV	73.9
	48	F	IV	72.4
	48	F	IV	80.2
	71	M	III	54.4

Supplementary Table 3. Primers for quantitative real time polymerase chain reaction (qRT-PCR)

Primer	Type	Primer sequence (5′-3′)
Col2a1	Forward	GAGCGGAGACTACTGGATTGA
	Reverse	TCTGGACGTTAGCGGTGTT
Acan	Forward	TTCCACCAGTGCGATGCAG
	Reverse	TGGTGTCCCGGATTCCGTA
MMP13	Forward	CAGCCCTATCCCTTGATGCCATTAC
	Reverse	GGGTGCAGACGCCAGAAGAATC
ADAMTS5	Forward	TCCTCTTGGTGGCTGACTCTTCC
	Reverse	TGGTTCTCGATGCTTGCATGACTG
β-actin	Forward	CGTGCGTGACATCAAAGAGAAG
	Reverse	CGTTGCCAATAGTGATGACCTG

Supplemental References

1. Wang X. D., Kou X. X., He D. Q., et al. (2012). Progression of cartilage degradation, bone resorption and pain in rat temporomandibular joint osteoarthritis induced by injection of iodoacetate. *PLoS One* **7**:e45036. DOI:10.1371/journal.pone.0045036
2. Zhang K., Yu J., Li J., et al. (2024). The Combined Intraosseous Administration of Orthobiologics Outperformed Isolated Intra-articular Injections in Alleviating Pain and Cartilage Degeneration in a Rat Model of MIA-Induced Knee Osteoarthritis. *Am J Sports Med* **52**:140-154. DOI:10.1177/03635465231212668
3. Bi R., Yin Q., Mei J., et al. (2020). Identification of human temporomandibular joint fibrocartilage stem cells with distinct chondrogenic capacity. *Osteoarthritis Cartilage* **28**:842-852. DOI:10.1016/j.joca.2020.02.835
4. Jiang Y. and Tuan R. S. (2015). Origin and function of cartilage stem/progenitor cells in osteoarthritis. *Nat Rev Rheumatol* **11**:206-212. DOI:10.1038/nrrheum.2014.200
5. Zhou C., Liu S., Li J., et al. (2018). Collagen Functionalized With Graphene Oxide Enhanced Biomimetic Mineralization and in Situ Bone Defect Repair. *ACS Appl Mater Interfaces* **10**:44080-44091. DOI:10.1021/acsami.8b17636
6. Liu H., Tian Y., Xue C., et al. (2022). Analysis of extracellular vesicle DNA at the single-vesicle level by nano-flow cytometry. *J Extracell Vesicles* **11**:e12206. DOI:10.1002/jev2.12206
7. Suades R., Greco M. F., Padro T., et al. (2025). Blood CD45(+)/CD3(+) lymphocyte-released extracellular vesicles and mortality in hospitalized patients with coronavirus disease 2019. *Eur J Clin Invest* **55**:e14354. DOI:10.1111/eci.14354
8. Valente A. J., Maddalena L. A., Robb E. L., et al. (2017). A simple ImageJ macro tool for analyzing mitochondrial network morphology in mammalian cell culture. *Acta Histochem* **119**:315-326. DOI:10.1016/j.acthis.2017.03.001
9. Suh J., Kim N. K., Shim W., et al. (2023). Mitochondrial fragmentation and donut formation enhance mitochondrial secretion to promote osteogenesis. *Cell Metab* **35**:345-360 e347. DOI:10.1016/j.cmet.2023.01.003
10. Aretz I., Hardt C., Wittig I., et al. (2016). An Impaired Respiratory Electron Chain Triggers Down-regulation of the Energy Metabolism and De-ubiquitination of Solute Carrier Amino Acid Transporters. *Mol Cell Proteomics* **15**:1526-1538. DOI:10.1074/mcp.M115.053181

11. Zheng C., Sui B., Zhang X., et al. (2021). Apoptotic vesicles restore liver macrophage homeostasis to counteract type 2 diabetes. *J Extracell Vesicles* **10**:e12109. DOI:10.1002/jev2.12109
